# Metabolic profiles show few differences in serum amino acid, one-carbon, and fatty acid compounds in dogs fed a plant-based (“vegan”) or meat-based diet

**DOI:** 10.3389/fvets.2025.1706965

**Published:** 2026-06-09

**Authors:** Sarah A. S. Dodd, Jennifer L. Adolphe, Anna K. Shoveller, Cate Dewey, Deep Khosa, David W. L. Ma, Sarah K. Abood, Adronie Verbrugghe

**Affiliations:** 1 Department of Population Medicine, Ontario Veterinary College, University of Guelph, Guelph, ON, Canada; 2 Department of Veterinary Biomedical Sciences, University of Saskatchewan, Saskatoon, SK, Canada; 3 Petcurean Pet Nutrition, Chilliwack, BC, Canada; 4 Department of Animal Biosciences, University of Guelph, Guelph, ON, Canada; 5 Department of Human Health and Nutritional Sciences, University of Guelph, Guelph, ON, Canada; 6 Department of Clinical Studies, Ontario Veterinary College, University of Guelph, Guelph, ON, Canada

**Keywords:** canine nutrition, circulating metabolites, lipid metabolism, plant-based ingredients, protein metabolic characteristic, vegan dog

## Abstract

**Introduction:**

Dogs are omnivores, not herbivores, and yet entirely plant-based diets are formulated to meet their current known nutrient recommendations. However, little is known about the metabolic effects of feeding diets containing no animal-derived nutrients. Metabolomics allows for the investigation of dietary influences on animal metabolism and physiology beyond what may be revealed by routine healthcare assessments.

**Methods:**

This study compared serum metabolomics in a longitudinal trial involving 61 healthy adult dogs fed an experimental extruded plant-based (“vegan”) diet (PLANT, *n* = 31) or a commercial extruded meat-based diet (MEAT, *n* = 30) for 3 months. Both diets met industry nutrient recommendations for maintenance of adult dogs and had similar levels of total protein and fat, though amino acid and fatty acid profiles differed. Free amino acid and protein, one-carbon, carbohydrate, fatty acids, and lipid metabolites were analyzed at the start and end of the trial. Organic acids and lipid metabolites were measured using direct injection liquid chromatography tandem mass spectrometry, one-carbon, and folate pathway metabolites by liquid chromatography-multiple reaction monitoring mass spectrometry, and fatty acids by gas chromatography. Repeated measures mixed modeling assessed metabolite differences between diet groups over time.

**Results:**

In total, 11 of 47 amino acid and protein metabolites, 0 of 16 carbohydrate metabolites, 3 of 29 one-carbon/folate pathway metabolites, 21 of 61 fatty acids, and 27 of 78 lipid metabolites differed between diets. Dogs maintained on PLANT demonstrated few changes associated with sulfur amino acid metabolism, but unexpectedly showed a reduction in the serum branched-chain amino acids (BCAA) isoleucine and valine despite higher dietary provision, though no differences in BCAA ketoacids were found. A reduction in serum creatinine without corresponding changes in creatine was also observed in the PLANT group, along with a lower total serum FA and omega-6 to omega-3 FA ratio, despite higher fat content in PLANT. In summary, dogs fed PLANT for 3 months showed few metabolic alterations compared with dogs fed MEAT; those detected were mostly attributable to differences in dietary composition.

**Discussion:**

This study demonstrated a few differences in nutrient metabolism attributable to the presence or absence of animal products in the diet over 3 months in adult dogs. Based on these findings, further research is warranted using plant-based diets of varying nutritional composition to determine longer-term physiological impacts and how these may affect and be affected by different life stages and health outcomes.

## Introduction

Dogs are omnivores, capable of consuming, digesting, absorbing, and utilizing nutrients from both plant and animal sources (1, 2). However, to the best of the authors' knowledge, the metabolic effects of feeding a strictly plant-based (“vegan”) diet to dogs have not yet been described. Indeed, few studies investigating the effects of plant-based diets on metabolism and physiology and their impacts on the health and wellbeing of dogs have been published. One diet trial demonstrated comparable hematological parameters between dogs fed experimental meat-free or meat-containing diets for 4 months (3), while another found comparable hematologic, biochemical, and echocardiographic parameters in dogs fed a commercial plant-based diet for 3 months (4). In contrast, when the nutrient composition of plant-based diets intended for feeding to dogs has been investigated, the provision of some essential nutrients was not adequate relative to canine nutrient requirements in a number of products, despite the availability of non-animal sources of these nutrients (5–8). It must be noted that this is not a problem exclusive to plant-based diets, as nutrient imbalances and insufficiencies have also been demonstrated in conventional meat-based diets (9, 10). Nevertheless, particular concern has been raised regarding the possibility of insufficient provision and/or digestibility of indispensable amino acids from plant-based proteins, especially the dietary limiting sulfur amino acids, potentially contributing to sub-normal production or retention of taurine (11–13). Though taurine is not recognized as a dietary essential nutrient for dogs, it is possible that insufficient provision of sulfur amino acids, poor protein digestibility, amino acid bioavailability, and/or reduced taurine-conjugated bile acid recycling may be potential dietary factors that may contribute to taurine deficiency and its clinical manifestation of dilated cardiomyopathy (DCM) (14, 15). Other associations between plant ingredients and DCM have also been postulated, suggesting potentially detrimental metabolites, as opposed to nutrient deficiencies (16). Furthermore, fatty acid, calcium, phosphorus, and vitamin D insufficiencies have also been demonstrated in plant-based diets (5). The authors investigated vitamin D and bone mineral content as part of this diet trial, with the results published elsewhere (17).

Metabolomics is the study of small molecules, metabolites, combining analytical chemistry with bioinformatic tools to detect patterns in metabolic profiles (18). Circulating metabolite concentrations represent the combined effects of nutrient intake and absorption, metabolic activity of gut microbiota, gene expression, and tissue metabolism in the body of the animal from whom it is sampled (18, 19). In dogs, serum metabolomic studies have included descriptive investigations (20, 21), comparative studies between dogs with or without health conditions (22–28), and studies to determine the effects of different dietary interventions (29–35).

Given the ongoing investigations into the suitability of some diets, including plant-based diets, for canine maintenance and concerns regarding potential association with DCM (36), it was hypothesized that differences in protein and amino acid metabolic pathways might be detected. Particularly, increased endogenous flux of sulfur amino acids to taurine synthesis was expected in the dogs fed the plant-based diet. Given the role of the essential amino acid methionine as a methyl group (single carbon) donor, should sulfur amino acid metabolism be altered, this could also have possible implications on one-carbon (1C) metabolism, and/or vice versa (37). One-carbon metabolism refers to biochemical pathways involving the transfer of single carbon units and is critical for the synthesis of proteins and nucleotides, amino acid homeostasis, and epigenetic control of DNA expression. Fatty acids and lipid metabolites were anticipated to respond to the fatty acid composition of the diets, as has been found using conventional canine diets (38, 39). The objective of this study was to compare the fasted serum metabolome of dogs fed either a plant-based or animal-based extruded diet formulated for adult maintenance to determine if metabolic alterations potentially associated with adverse health outcomes were detectable.

## Methodology

This study was conducted at the University of Guelph with the approval of the Research Ethics Board (Research Ethics Approval number 19-02-036) and Animal Care Committee (Animal Use Protocol #4129), ensuring the research protocol was in line with institutional, provincial, and national guidelines and policies for humans participating in research as well as for the care and use of animals in research. Samples for this study were collected as part of a larger prospective longitudinal study investigating the effects of a plant-based (PLANT, i.e., “vegan”) vs. an animal-based (MEAT) diet over a 3-month period.

Sample size determination was performed based on previous nutrition studies using similar numbers of dogs and justified with calculations based on the average bone mineral density of dogs as part of a complementary study (17). Participants were enrolled using an eSurvey designed on the Qualtrics (Provo, Utah, USA) platform to collect data regarding patient suitability (17). Dogs were excluded from consideration if they were intact, weighed less than 5 kg, had an owner-reported body condition score (BCS) > 5, fed a homemade or raw diet, housed outdoors without supervision, had access to unmonitored food sources, had current medical problems, received medication other than parasite preventatives, had previous medical problems that could affect them currently, or had known dietary allergies. Recruited dogs were brought to the University of Guelph's Ontario Veterinary College for an enrolment appointment conducted by a licensed veterinarian (author SD). Following the collection of medical and dietary history, physical examination was performed, body weight (BW) was measured, and blood was collected for analysis of complete blood count and serum biochemistry. The same veterinarian attended each dog at each (enrolment, baseline, and exit) appointment. Description of the enrolled dogs is available elsewhere (17).

Two isocaloric diets, one commercial diet^1^ containing animal-derived ingredients (MEAT) and one experimental “vegan” diet containing no animal-derived ingredients (PLANT), formulated to meet or exceed nutrient recommendations for adult maintenance (40) were manufactured in small batches under similar conditions in the same facility. When ingredients were shared between the two diets, the same batches of ingredients were used. The diets were packaged into identical sealed white bags and labeled prior to distribution to participants. The investigators and participants were blinded to the identity of the diets. Diet identities were kept by a third person employed at the University of Guelph, who was not involved in data collection, statistical analysis, and data interpretation, until statistical analyses were complete. Nutrient analyses, including protein and amino acids, fat and fatty acids, fiber, B vitamins, and choline, were performed on the diets post-manufacturing [Table 1, see also Dodd et al. (17) for mineral and fat-soluble vitamin analyses and Liversidge et al. (41) for fiber analyses]. For amino acid analyses, samples underwent hydrolysis, oxidized hydrolysis, or alkaline hydrolysis before amino acid profiling was performed by ultra-performance liquid chromatography, as described by Cargo-Froom et al. (42). Fatty acids were extracted by organic solvent in chloroform and methanol, then fatty acids were saponified and methylated using boron trifluoride prior to analysis by gas chromatography with C17:0 as an internal standard for quantification, as described elsewhere (43). Vitamins were analyzed by third-party contract laboratories as previously described (17).

**Table 1:** Selected nutrient composition (on a dry matter basis) of commercial animal-based diet (MEAT) and experimental plant-based diet (PLANT) fed to client-owned dogs for 3 months.

**Nutrient**	**MEAT**	**PLANT**
Total protein (g/100g)	29.48	25.41
Arginine (g/100g)	2.10	1.92
Cystine (g/100g)	0.83	0.44
Histidine (g/100g)	0.65	0.67
Isoleucine (g/100g)	1.00	1.21
Leucine (g/100g)	2.05	2.23
Lysine (g/100g)	1.64	1.57
Methionine (g/100g)	0.79	0.61
Phenylalanine (g/100g)	1.28	1.62
Threonine (g/100g)	1.14	1.19
Tryptophan (g/100g)	0.16	0.15
Tyrosine (g/100g)	0.61	0.58
Valine (g/100g)	1.31	1.50
Taurine (g/100g)	0.25	0.09
Total fat (g/100g)	14.03	15.99
Total omega-6 fatty acids (g/100g)	3.11	2.06
Total omega-3 fatty acids (g/100g)	0.40	0.58
Eicosapentaenoic acid [EPA] (g/100g)	0.00	0.00
Docosahexaenoic acid [DHA] (g/100g)	0.01	0.01
Nitrogen-free extract (g/100g)^*^	47.93	51.07
Crude fiber (g/100g)	3.61	4.18
Total dietary fiber (g/100g)	9.6	15.5
Insoluble fiber (g/100g)	7.5	4.6
Soluble fiber (g/100g)	2.1	10.9
Resistant starch (g/100g)	7.9	2.7
Thiamine (mg/kg)	14.2	14.6
Riboflavin (mg/kg)	18.2	13.4
Niacin (mg/kg)	77.5	80.8
Pantothenic acid (mg/kg)	53.5	31.8
Pyridoxine (mg/kg)	6.49	6.24
Folic acid (mg/kg)	0.96	1.30
Cobalamin (mg/kg)	0.07	0.06
Choline (mg/kg)	1,322	2,323
^*^Nitrogen-free extract is an approximation of carbohydrate content, calculated by subtraction of the sum of crude protein, crude fat, crude fiber, moisture, and ash from a total of 100 (40).		
**PLANT ingredients**		
Peas, barley, oats, potato protein, sunflower oil (preserved with mixed tocopherols), pea protein, lentils, quinoa, calcium carbonate, dicalcium phosphate, primary dried yeast, flaxseed, natural vegetable flavoring, salt, dried marine algae, choline chloride, vitamins (vitamin A supplement, vitamin D2 supplement, vitamin E supplement, niacin, L-ascorybyl-2-polyphosphate (a source of vitamin C), d-calcium pantothenate, thiamine mononitrate, riboflavin, pyridoxine hydrochloride, folic acid, biotin, and vitamin B12 supplement), minerals (zinc proteinate, iron proteinate, copper proteinate, zinc oxide, manganese proteinate, copper sulfate, ferrous sulfate, calcium iodate, manganous oxide, and selenium yeast), DL-methionine, potassium chloride, L-lysine, taurine, L-carnitine, and dried rosemary		
**MEAT ingredients**		
Chicken meal, de-boned chicken, whole brown rice, white rice, oatmeal, chicken fat (preserved with mixed tocopherols), potatoes, salmon meal, natural chicken flavor, whole dried egg, flaxseed, pea fiber, alfalfa, apples, carrots, cranberries, sodium chloride, potassium chloride, dried chicory root, dried Lactobacillus acidophilus fermentation product, dried *Enterococcus faecium* fermentation product, vitamins (vitamin A supplement, vitamin D3 supplement, vitamin E supplement, niacin, L-ascorbyl-2-polyphosphate (a source of vitamin C), d-calcium pantothenate, thiamine mononitrate, beta-carotene, riboflavin, pyridoxine hydrochloride, folic acid, biotin, and vitamin B12 supplement), minerals (zinc proteinate, iron proteinate, copper proteinate, zinc oxide, manganous proteinate, copper sulfate, ferrous sulfate, calcium iodate, manganous oxide, and selenium yeast), DL-methionine, L-lysine, taurine, yucca schidigera extract, and dried rosemary.		

Food quantity was calculated based on the dog's current dietary intake to match calories and maintain current BW, and a gram scale was provided to precisely measure out the recommended quantity of food per day, as well as any leftovers. Participants were instructed not to feed their dogs any other food for the duration of the study, with exceptions for some pieces of fruit or vegetables or plant-based treats, which were required to be recorded. Participants were given a list of treats that could be fed for the duration of the study (plant-based treats, without added micronutrients). An acceptable treat dose was given for each dog to avoid exceeding 10% of their daily energy intake from sources other than the trial diet.

An initial adaptation period of 4 weeks was performed between the screening and baseline appointments to ensure all dogs started the trial on the same diet (MEAT) and mitigate variation due to differences in pre-trial diets. Upon completion of the 4-week adaptation phase, the dogs were scheduled for baseline appointments and randomly allocated to treatment groups (MEAT or PLANT) using a virtual random number generator (Google LLC, Alphabet Inc., California, USA) prior to the baseline evaluation. Baseline evaluations consisted of a veterinary wellness examination (medical and dietary history, physical examination, complete blood count, serum biochemistry, and urinalysis), sedation, and body composition analysis by dual-energy x-ray absorptiometry [see Dodd et al. (17) for body composition outcomes, and blood collection for serum metabolomics].

After the baseline evaluation, dogs changed to their respective experimental diet—either continuing MEAT or starting PLANT. Dogs were fed the trial diets for 12 weeks, then returned at the end of the trial for their exit evaluation. Exit evaluations were the same as the baseline evaluations. Throughout the study, including the 4-week adaptation and the 12-week experimental period, participants were asked to record the quantity of food offered, the quantity of food eaten, the amount and type of snacks or treats provided, frequency of defecation, fecal condition score, BCS, BW, duration of walks or play activity, and any other notable events in a daily diary. Owners were asked to weigh their dogs on scales. Fecal condition score and BCS charts were provided (44, 45). A depiction of the trial timeline and description of the impacts of the global COVID-19 pandemic have been published previously (17). Briefly, seven dogs were lost to follow-up during the pandemic, while the length of the trial was elongated for 10 dogs as exit timepoint analyses were delayed.

At the baseline and exit timepoints, blood was collected from the saphenous or jugular vein, depending on dog size, using a 22G BD Vacutainer^®^ needle (Becton, Dickinson and Company, Franklin Lakes, New Jersey, USA) after an overnight fast. Blood was collected for serum metabolomics into BD red top Vacutainer^®^ blood collection tubes (Becton, Dickinson and Company, Franklin Lakes, New Jersey, USA) with no additives. After clotting for 30 min at room temperature, serum was separated from whole blood in plain red top tubes by centrifugation at 1,500 G in a refrigerated centrifuge set to 4 °C. Sera aliquots were immediately frozen at −80 °C in 1.5 mL Eppendorf tubes and stored until analyzed. All samples were batched once sample collection was complete.

Detection of organic acids and their derivatives, lipids, and lipid-like molecules was performed by direct injection (DI) liquid chromatography (LC) tandem mass spectrometry (MS/MS) “TMIC PRIME” assay at the Metabolomics Innovation Centre laboratory at the University of Alberta, as previously described elsewhere (46). A 96-well deep plate with a filter plate attached with sealing tape was used. First, 14 wells were used for one blank, three zero samples, seven standards, and three quality control samples. For all metabolites except organic acid, samples were thawed on ice and vortexed, and centrifuged at 13,000× g. Ten μL of each sample was loaded onto the center of the filter on the upper 96-well plate and dried in a stream of nitrogen. Subsequently, phenyl-isothiocyanate was added for derivatization. After incubation, the filter spots were dried again using an evaporator. Extraction of the metabolites was then achieved by adding 300 μL of extraction solvent. The extracts were obtained by centrifugation into the lower 96-deep well plate, followed by a dilution step with mass spectrometry (MS) running solvent. For organic acid analysis, 150 μL of ice-cold methanol and 10 μL of isotope-labeled internal standard mixture were added to 50 μL of the sample for overnight protein precipitation. Then it was centrifuged at 13,000× g for 20 min. Furthermore, 50 μL of supernatant was loaded into the center of wells of a 96-deep well plate, followed by the addition of 3-nitrophenylhydrazine reagent. After incubation for 2 h, butylated hydroxytoluene stabilizer and water were added. Next, targeted quantitative analysis of samples was performed using a combination of DI MS with a reverse-phase LC–MS/MS custom assay. Mass spectrometric analysis was performed on an ABSciex 4000 Qtrap^®^ tandem MS instrument (Applied Biosystems/MDS Analytical Technologies, Foster City, CA) equipped with an Agilent 1,260 series ultra-high performance (UP) LC system (Agilent Technologies, Palo Alto, CA). The samples were delivered to the mass spectrometer by an LC method followed by a DI method. A total of 143 metabolites were screened, and data analysis was done using Analyst 1.6.2. The 143 measured analytes included acylcarnitines, amino acids, biogenic amines, fatty acids, lipids, lysophosphatidylcholines, neurotransmitters, organic acids, phosphatidylcholines, sphingomyelins, and sugars (see Supplementary Table 1 for the full list of PRIME metabolites).

Detection of 311C and folate pathway metabolites was performed by LC-multiple reaction monitoring (MRM)/MS “TMIC One-carbon and Folate Metabolism Pathway” at the Metabolomics Innovation Centre laboratory at the University of Victoria. See Supplementary Table 2 for the full list of 1C and folate pathway metabolites. For cobalamin, folate, s-adenosylmethionine, s-adenosylhomocysteine (SAH), folate, and derivatives, samples were thawed on ice, then 50 μL of serum was mixed with 50 μL of internal standard solution (containing SAH-D4, 200-mM ammonium formate, and 10 g/L ascorbic acid) and vortexed. In total, 150 μL of acetonitrile was added, then the samples were vortexed again, followed by sonication in an ice-water bath for 1 min, prior to centrifugation at 21,000 g for 15 min. Supernatant was collected and then dried under nitrogen, and then the residue was resuspended in 50 μL of 10% methanol. After centrifugation, 10 μL of the supernatant and standard solutions containing reference standards of the targeted compounds were injected into a Waters Acquity (Milford, Massachusetts, USA) UPLC system coupled to a Sciex (Marsiling, Singapore) XTRAP 65,000 Plus mass spectrometer operated in the positive-ion mode. Liquid chromatography separation was performed on a C18 UPLC column (2.1 × 100 mm, 1.8 μm) with 0.1% formic acid in water (A) and 0.1% formic acid in acetonitrile (B) as the binary solvents for gradient elution (2% to 25% B in 12 min) at 40 °C and 0.3 mL/min. Concentrations of the detected metabolites were calculated with internal standard calibration by interpolating the constructed linear-regression curves of individual compounds with the analyte to internal standard peak ratios measured from each sample solution.

For taurine and homoserine, samples were thawed on ice, then 10 μL were mixed with 25 μL of acetonitrile. The mixture was vortexed, then sonicated for 1 min in an ice-water bath, followed by centrifugation at 21,000 g at 5 °C for 15 min. A total of 20 μL of supernatant or standard solution (0.0005–100 μM reference standards of targeted compounds) was mixed with 20 μL of the internal standard solution (containing 13C or deuterium-labeled targeted amino acids), 40 μL of 20 mM dansyl chloride solution, and 40 μL of pH = 9 borate buffer. The mixtures were allowed to react at 40°C for 30 min, prior to mixing with 480 μL of 50% acetonitrile. Then, 10 μL aliquots of each resultant solution and each standard solution were injected into a Waters Acquity (Milford, Massachusetts, USA) UPLC system coupled to a Sciex (Marsiling, Singapore) QTRAP 4,000 mass spectrometer operated in the positive-ion mode. LC separation was carried out on a C18 UPLC column (2.1 × 150 mm, 1.8 μm) with 0l.1% formic acid in water (A) and 0.1% formic acid in acetonitrile/isopropanol (B) as the binary solvents for gradient elution (20% A to 50% B in 10 min) at 55 °C and 0.3 mL/min. Concentrations of the detected metabolites were calculated with internal standard calibration by interpolating the constructed linear-regression curves of individual compounds with the analyte to internal standard peak ratios measured from each sample solution.

For free glutathione, homocysteine, betaine, and choline, samples were thawed on ice, then 15 μL was mixed with 35 μL of acetonitrile and vortexed, then sonicated for 10 s in an ice-water bath. Samples were centrifuged at 21,000 g at 5 °C for 15 min, then 20 μL of the supernatant was mixed with 180 μL of the internal standard solution (containing isotope-labeled glutathione, homocysteine, betaine, and choline in 25-mM N-ethylmaleimide). Furthermore, 10 μL aliquots of the resultant solutions and each standard solution were injected into an Agilent (Waldbronn, Germany) 1290 UPLC system coupled to an Agilent 6495B QQQ mass spectrometer operated in the positive-ion mode. Liquid chromatography separation was carried out on a C18 UPLC column (2.1 × 100 mm, 1.9 μm) with a 5-mM ammonium acetate solution (A) and methanol (B) as the binary solvents for gradient elution (0% A to 60% B in 15 min) at 45 °C and 0.25 mL/min. Concentrations of the detected metabolites were calculated with internal standard calibration by interpolating the constructed linear-regression curves of individual compounds with the analyte to internal standard peak ratios measured from each sample solution.

In total, 67 fatty acids were extracted by organic solvent in chloroform and methanol, then saponified and methylated using boron trifluoride. Methylated FAs were separated and analyzed by gas chromatography, as described in Burns et al. (47). C17:0 was added as an internal standard for quantification. See Supplementary Table 3 for the full list of free FAs.

Prior to statistical analysis, metabolites were categorized as protein and amino acid metabolites, lipid and lipid-like metabolites, and/or carbohydrate and TCA cycle metabolites, based on descriptions of each compound available via MetaboAnalyst^2^ pathway analysis, the Metabolomics Innovation Centre Human Metabolome Database,^3^ and the National Library of Medicine PubChem^4^ platforms. Some metabolites were included in more than one category, and some metabolites were measured in duplicate, being included both in the PRIME and 1-C analytical packages (see Supplementary Figures 1–9 for Bland-Altman plots demonstrating agreement between analytical methods).

All analyses were performed using commercial statistical software (StataIC, StataCorp, College Station, Texas, USA, and MetaboAnalyst 5.0 (Montreal, Canada), http://www.metaboanalyst.ca). Independent variables (dog sex, age, weight, and BCS) were tested for normality of distribution using the Shapiro-Wilk normality test and visual evaluation of frequency histograms and normal probability plots. Differences in the distribution of independent variables (age, sex, and BCS) between diet groups after randomization were tested using the *t*-test for parametric and the Wilcoxon rank-sum test for non-parametric data.

Dependent variables (metabolites) were tested for normality of distribution using the Shapiro-Wilk normality test and visual evaluation of frequency histograms and normal probability plots. Non-normally distributed variables were log-transformed prior to analyses. Differences in metabolites between dogs of different age, sex, and BCS were tested using ANOVA and *post-hoc* Tukey test. Effects between diets over time were investigated via repeated measures mixed model analyses using residual maximum likelihood, with dog ID as the variable on which data were repeated. For some variables (leucine, tryptophan, TMAO, and THF), there were differences detected between groups at baseline; thus, baseline was included as a covariate in all analyses. *Post hoc* analyses were performed by contrasting main effects and graphing the interactions. As multiple comparisons were made, the critical cut-off for *P-values* was adjusted to control for a false discovery rate of less than or equal to 10% according to the Benjamini–Hochberg procedure, as recommended for bioinformatics data (48).

Data with significant associations detected on repeated measures mixed model were uploaded to the MetaboAnalyst^b^ platform and normalized for comparison by row-wise normalization (median or quantile normalization), mathematical transformation (log or square root), and/or data scaling (autoscaling) as appropriate. Visual inspection of density plots and box plots was performed to evaluate the normalization technique. An overview of the data was visualized using interactive principal component analysis, and two-way heatmaps were generated with Euclidean distance measure and the Ward clustering algorithm.

## Results

The independent variables considered for each group were sex, age, BW, BCS, and the seasons during which the dog was participating in the study. After randomization, their distribution of independent variables between groups did not differ (17). Of 87 potential dog participants, 76 were enrolled in the study, but two were excluded due to abnormalities on entry laboratory analyses (one with elevated liver enzymes, the other with hypercalcemia). In total, 74 dogs started the trial, but 4 left the study during the control diet phase due to not liking the diet (1), excessive weight gain (1), or anal sac complications (2), leaving 70 dogs completing the control phase and baseline evaluations. Of these 70, an additional 9 dogs were lost due to lack of follow-up from the client (7), urinary tract infection (1), or complications from medication administration following injury (1). A total of 61 dogs completed the trial: 31 in the PLANT group and 30 in the MEAT group. Bodyweight did not differ between groups or between timepoints. Food calculated to match previous intake and maintain current bodyweight was recorded by pet owners and did not vary between groups or between timepoints. Furthermore, PLANT and MEAT diets were found to have comparable digestibility, and minimal differences in fecal microbiome or metabolome were found (41, 49, 50). Consumption of PLANT or MEAT for 3 months had no detectable effects on dog health and wellness as determined by physical examination, complete blood count, and serum biochemistry, and body composition did not differ (17).

### TMIC PRIME assay

A total of 143 serum metabolites were screened by DI/LC-MS/MS. In total, 11 (histamine, phenylethylamine, cis-hydroxyproline, dopamine, DOPA, carnosine, nitro-tyrosine, diacetylspermine, tyramine, phosphocreatine, and p-hydroxyhippuric acid) were excluded due to concentrations below the detection limits, leaving a total of 132 metabolites included for analyses.

**Figure 1:**
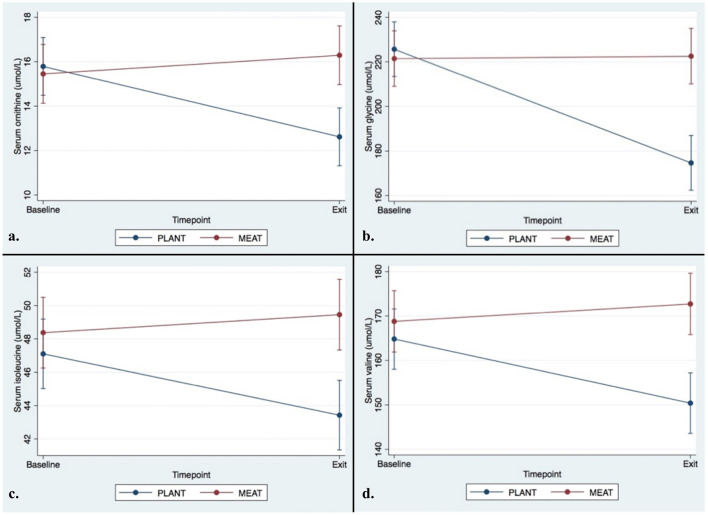
Serum amino acids **(a)** ornithine, **(b)** glycine, **(c)** isoleucine, **(d)** valine with significant differences between diet groups at the exit timepoint detected by repeated measures mixed modeling in 61 dogs fed an animal-based (MEAT) or plant-based (PLANT) diet for 3 months.

**Figure 2:**
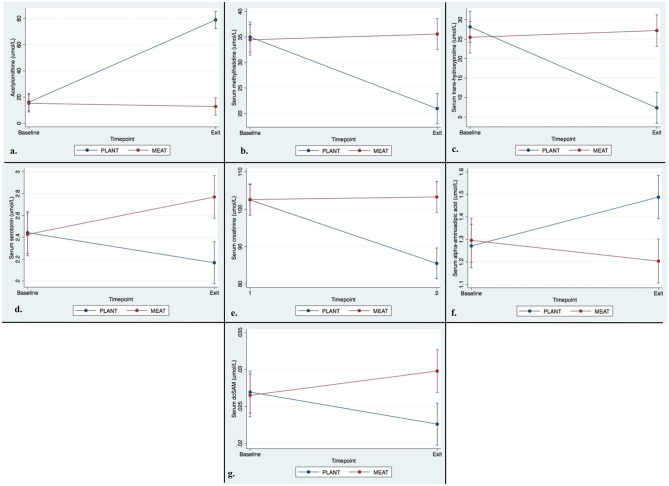
Serum protein and amino acid metabolites **(a)** acetylornithine, **(b)** methylhistidine, **(c)** trans-hydroxyproline, **(d)** serotonin, **(e)** creatinine, **(f)** alpha-aminoadipic acid, **(g)** dcSAM with significant differences between diet groups at the exit timepoint detected by repeated measures mixed modeling in 61 dogs fed an animal-based (MEAT) or plant-based (PLANT) diet for 3 months.

**Figure 3:**
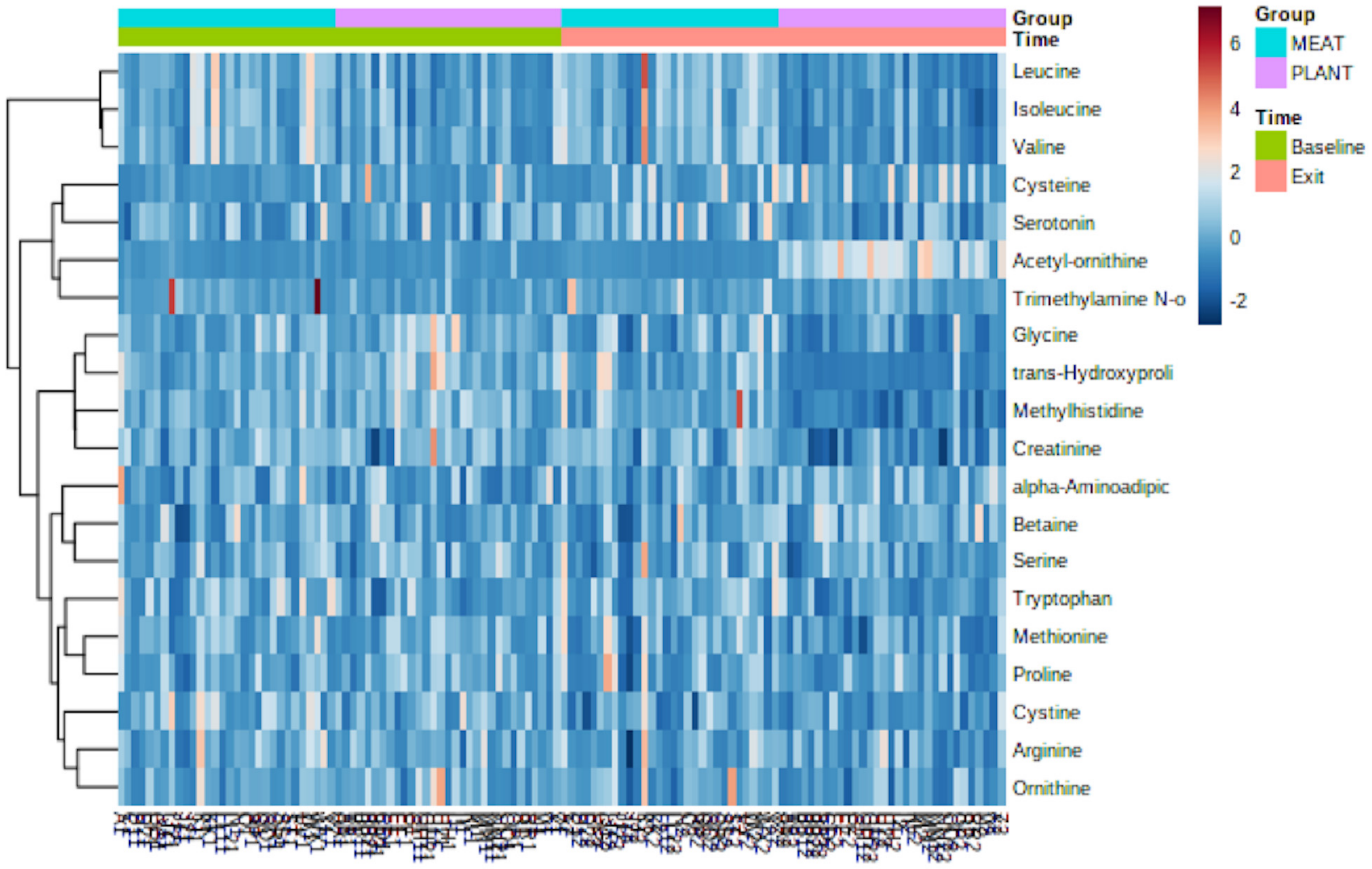
Two-way heatmap of significant serum amino acids and protein metabolites in 61 dogs fed an animal-based (MEAT) or plant-based (PLANT) diet for 3 months, based on repeated measures mixed modeling.

In total, 47 amino acids, amino acid derivatives, and protein metabolites were analyzed. Based on univariate mixed modeling, one amino acid (leucine) and one ammonium compound (TMAO) differed between diet groups as the baseline timepoint, while 18 amino acids and protein metabolites (acetylornithine, alpha-aminoadipic acid, arginine, creatinine, cysteine, cystine, dcSAM, glutamine, glycine, homocysteine, isoleucine, methionine, methylhistidine, ornithine, proline, serotonin, trans-hydroxyproline, and valine) were determined to be affected by time and/or treatment (Table 2, Supplementary Table 5). A total of four amino acids (ornithine, glycine, isoleucine, and valine) and seven protein metabolites (acetylornithine, methylhistidine, trans-hydroxyproline, serotonin, creatinine, alpha-aminoadipic acid, and dcSAM) were significantly different between diet groups at the exit timepoint (Figures 1, 2, respectively), most being lower in the PLANT group with the exception of acetylornithine and alpha-aminoadipic acid, which were higher. A heatmap was generated to visualize differences in amino acids and protein metabolites between the diet groups and timepoints (Figure 3).

**Table 2:** Significant differences in serum amino acids and protein metabolites in 61 dogs fed an animal-based (MEAT) or plant-based (PLANT) diet for 3 months.

**Metabolites (**μ**M)**		**Time (week)**	**MEAT (n30)**		**PLANT (n31)**		**P_Diet_**	**P_Time_**	**P_DietxTime_**
			**MSC**	**SEM**	**MSC**	**SEM**			
Amino acids	Arginine	0	145.17	4.12	141.43^A^	3.20	>0.1	0.011	>0.1
		12	140.35		133.49^B^				
	Cysteine	0	2.65^A^	0.39	3.46^A^	0.45	>0.1	0.015	>0.1
		12	2.93^B^		3.33^B^				
	Cystine	0	27.74^A^	0.98	23.36^A^	0.78	>0.1	0.001	>0.1
		12	26.66^B^		24.86^B^				
	Glutamine	0	722.67	15.32	735.48^A^	17.33	0.080	0.019	>0.1
		12	712.68		789.65^B^				
	Glycine	0	215.23	6.34	231.74^A^	6.75	0.020	>0.1	<0.001
		12	212.4^c^		180.71^Bd^				
	Isoleucine	0	49.96	1.20	45.57^A^	0.93	<0.001	>0.1	<0.001
		12	49.70^c^		41.90^Bd^				
	Leucine	0	174.97^c^	3.48	158.84^d^	2.44	0.020	0.100	>0.1
		12	125.04^c^		144.44^d^				
	Methionine	0	48.62	1.28	48.12^A^	1.05	0.100	0.005	>0.1
		12	47.86		43.80^B^				
	Ornithine	0	14.82	0.73	16.40^A^	0.73	>0.1	>0.1	<0.001
		12	15.00		13.23^B^				
	Proline	0	122.85	4.73	129.14^A^	3.37	>0.1	0.008	>0.1
		12	122.53		117.46^B^				
	Valine	0	174.97	4.21	158.84^A^	3.12	<0.001	>0.1	<0.001
		12	174.14^c^		144.44^Bd^				
Amino acid derivatives	Acetylornithine	0	15.06	0.81	16.33^A^	5.16	<0.001	0.100	<0.001
		12	13.63^c^		79.09^Bd^				
	alpha-AAA	0	1.33	0.07	1.23^A^	0.06	0.050	>0.1	<0.001
		12	1.27^c^		1.45^Bd^				
	Creatinine	0	102.71	1.92	102.41^A^	2.71	<0.001	>0.1	<0.001
		12	101.42^c^		85.37^Bd^				
	dcSAM	0	0.026	0.001	0.027^A^	0.001	0.024	0.100	<0.001
		12	0.027^c^		0.025^Bd^				
	Homocysteine	0	9.19^A^	0.19	9.19^A^	0.20	>0.1	<0.001	>0.1
		12	9.05^B^		9.09^B^				
	Methylhistidine	0	33.96	1.53	35.44^A^	1.34	<0.001	>0.1	<0.001
		12	33.99^c^		21.41^Bd^				
	Serotonin	0	2.39	0.13	2.48^A^	0.10	0.070	0.100	<0.001
		12	2.52		2.20^B^				
	trans-(OH)proline	0	23.97	1.82	29.63^A^	2.04	<0.001	>0.1	<0.001
		12	24.46^c^		8.83^Bd^				
Ammonium compounds	TMAO	0	4.65^c^	0.47	3.24^d^	0.16	0.047	>0.1	>0.1
		12	4.26^c^		3.37^d^				

MSC, mean serum concentration; dcSAM, S-adenosyl-methioninamine; alpha-AAA, alpha-Aminoadipicacid; trans-(OH)proline, Transhydroxyproline; TMAO, Trimethylamine N-oxide.

Rows or columns with no superscript indicates no significant differences for the measured parameter.

Down a column, different upper-case superscript letters (A, B) denote significant differences (*P* < 0.05) following a Tukey's *post hoc* analysis within groups (MEAT or PLANT) over time (week 0 to week 12).

Across a row, different lower-case letters (c, d) denote significant differences (*P* < 0.05) following a Tukey's *post hoc* analysis between groups (MEAT or PLANT) at one time point (week 0 or week 12).

In total, 16 carbohydrate and TCA cycle intermediates were analyzed. In both diet groups, alpha-ketoglutaric acid and succinic acid increased over time; repeated measures models did not identify any other significant effects for any carbohydrates and TCA cycle intermediates. Due to this dearth of significant effects, the data were not analyzed further.

**Figure 4:**
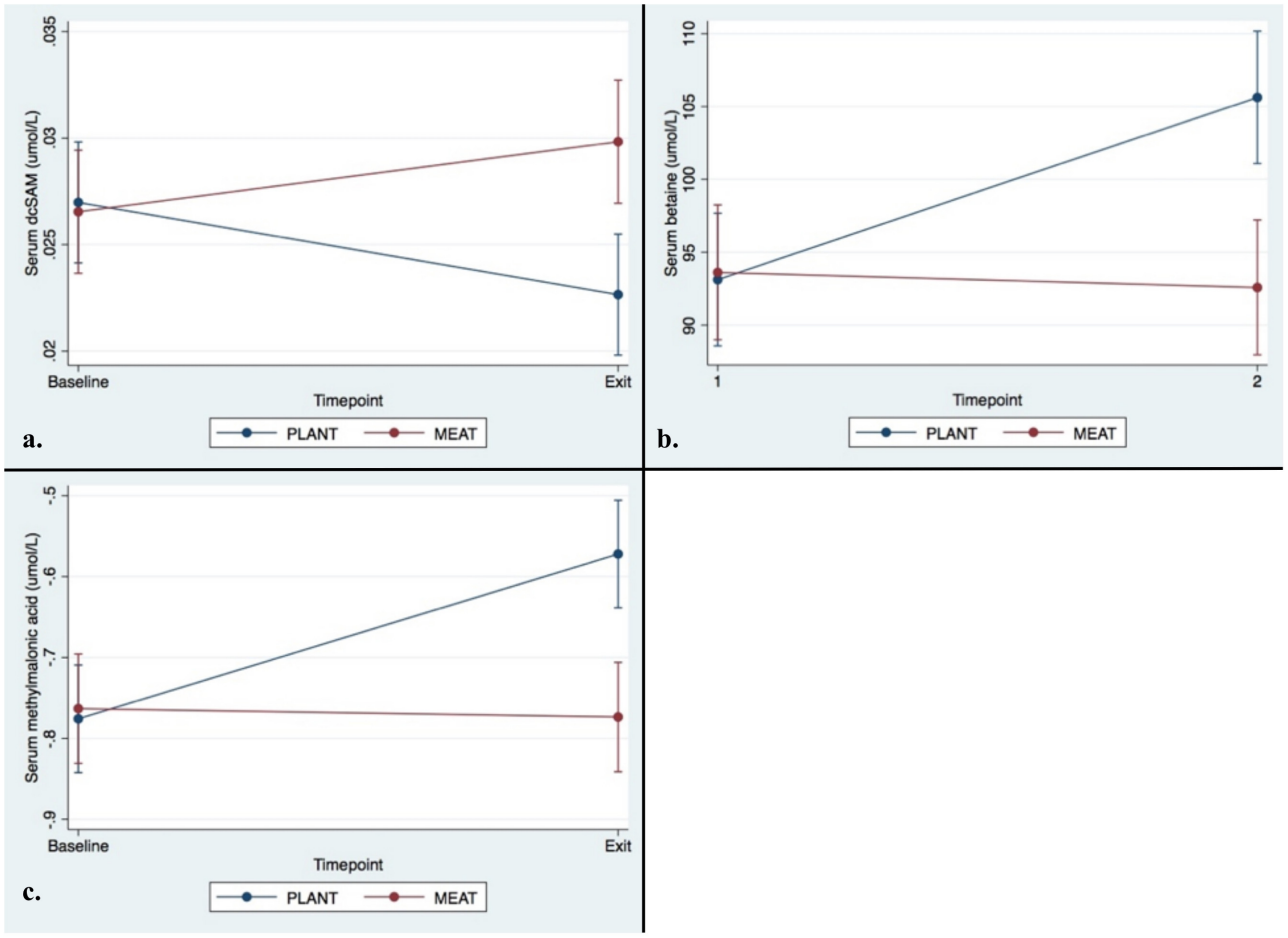
Folate pathway metabolites **(a)** dcSAM, **(b)** betaine, **(c)** methylmalonic acid with significant differences between diet groups at the exit timepoint detected by repeated measures mixed modeling in 61 dogs fed an animal-based (MEAT) or plant-based (PLANT) diet for 3 months.

**Figure 5:**
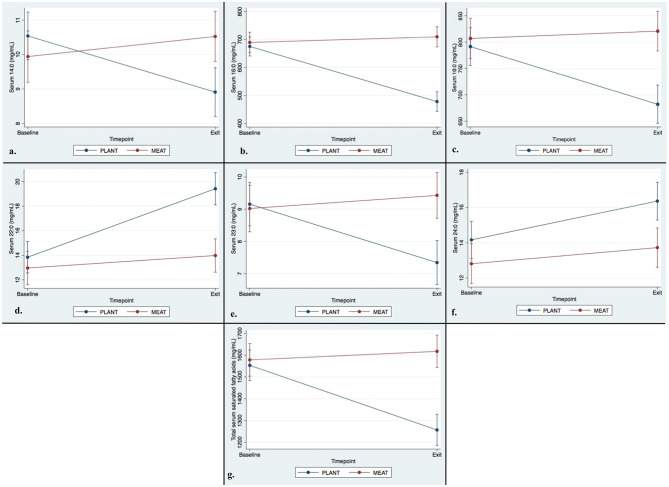
Saturated fatty acids **(a)** 14:0, **(b)** 16:0, **(c)** 18:0, **(d)** 22:0, **(e)** 23:0, **(f)** 24:0, **(g)** total with significant differences between diet groups at the exit timepoint, as detected by repeated measures mixed modeling in 61 dogs fed an animal-based (MEAT) or plant-based (PLANT) diet for 3 months.

**Figure 6:**
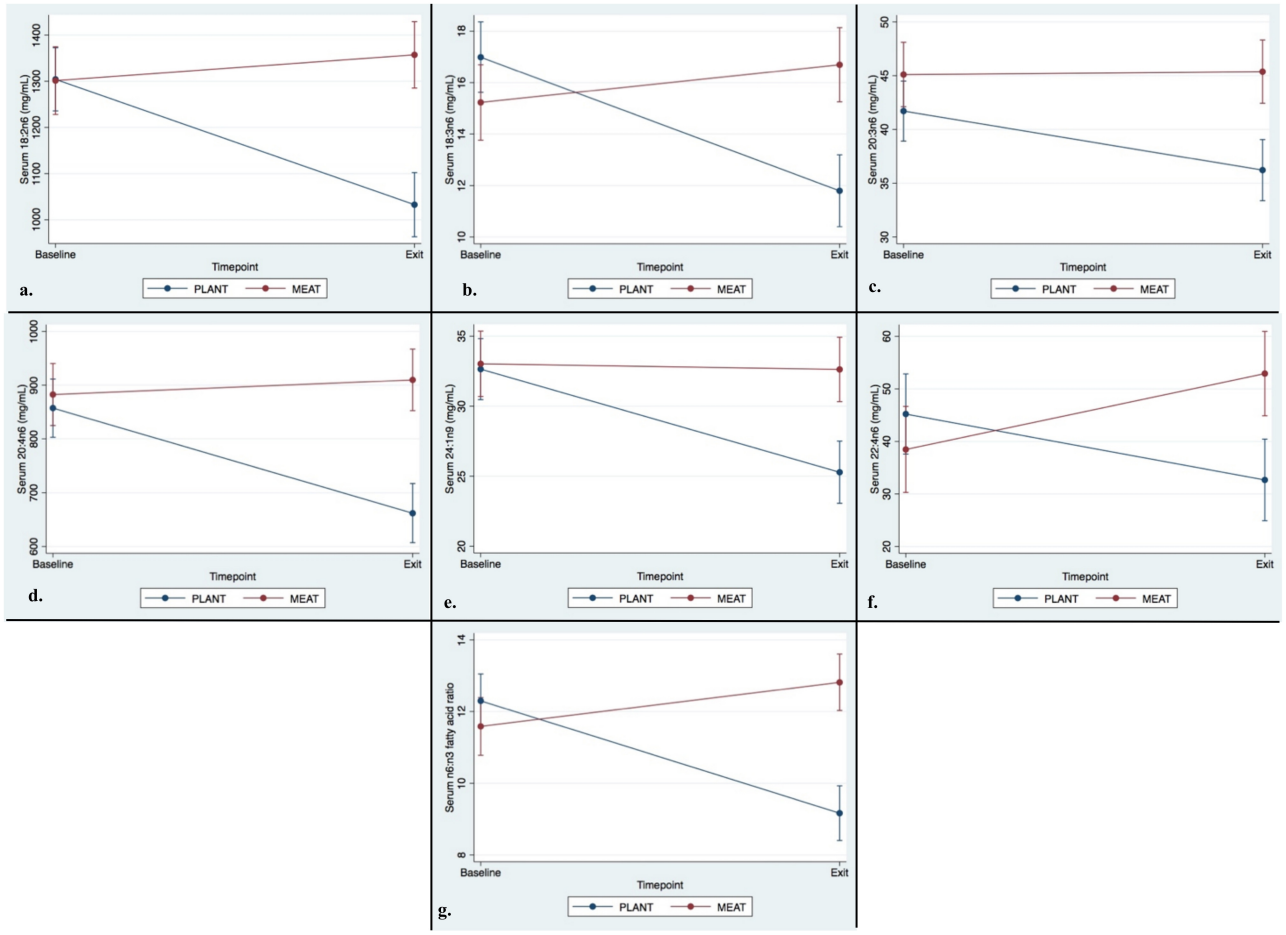
Polyunsaturated fatty acids **(a)** 18:2n6, **(b)** 18:3n6, **(c)** 20:3n6, **(d)** 20:4n6, **(e)** 24:1n9, **(f)** 22:4n6, **(g)** omega 6 to omega 3 ratio with significant differences between diet groups at the exit timepoint, as detected by repeated measures mixed modeling in 61 dogs fed an animal-based (MEAT) or plant-based (PLANT) diet for 3 months.

**Figure 7:**
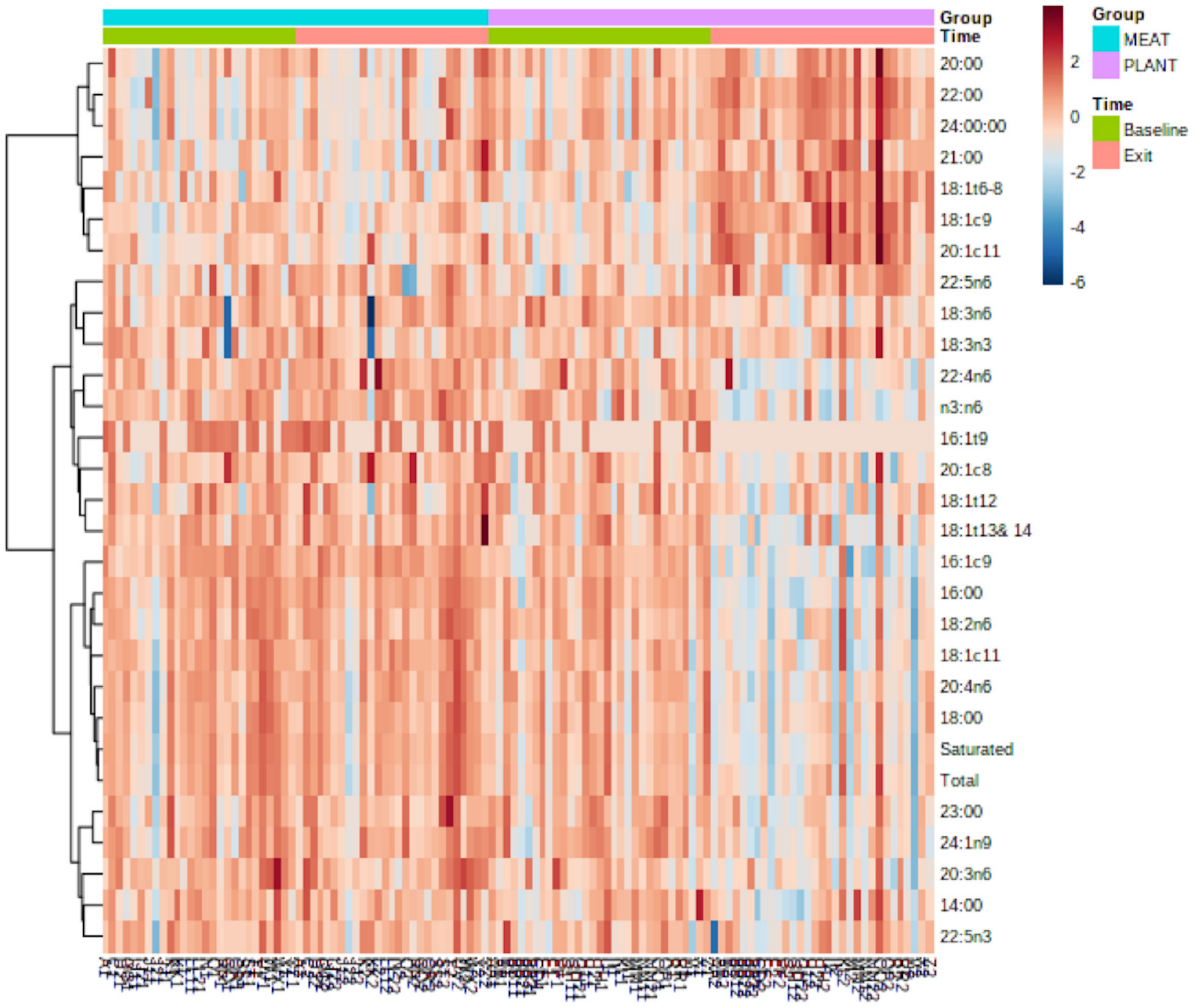
Two-way heatmap of significant fatty acids in 61 dogs fed an animal-based (MEAT) or plant-based (PLANT) diet for 3 months, based on repeated measures mixed modeling.

A total of 78 lipid metabolites and lipid-like molecules were measured. Based on univariate mixed modeling, eight PC (aa C32:2, aa C36:0, aa C 38:0, aa C40:1, aa C40:2, aa C40:6, ae 36:0, and ae 40:6), seven LPC (a C16:0, a C17:0, a C18:0, a C18:1, a C18:2, a C24:0, and C20:4), five sphingomyelins (SM) (C16:0, C16:1, C18:0, C18:1, and C20:2), five hydroxylated sphingomyelins (SMOH) (C14:1, C:16:1, C22:1, C22:2, and C24:1), carnitine (C0), and eight acylcarnitines [C2, C3, C4, C5, C14:1, C16, C16:1, and C16:2 (OH)] were determined to be affected (Table 3, Supplementary Table 6). Those differences between diet groups at the exit timepoint are depicted in Figures 4–6. Of the PCs, aa C36:0, aa C 38:0, aa C40:1, aa C40:2, aa C40:6, and ae 40:6 were higher in PLANT, while aa C32:2 and ae 36:0 were lower. Of the LPCs, a C17:0, a C18:1, and a C24:0 were higher in PLANT, while a C16:0 and a C20:4 were lower. Sphingomyelins C16:0, C16:1, C18:0, C18:1, and C20:2 were lower in PLANT. Hydroxylated SM C22:1, C22:2, and C24:1 were also lower. No SM or SMOH were significantly higher in PLANT at the exit timepoint. All lipid metabolites affected by time, diet, or the interaction of time and diet, as identified on mixed modeling, were included for further analyses using the MetaboAnalyst^b^ platform. A heatmap was generated to visualize differences between the diet groups and timepoints (Figure 7).

**Table 3:** Significant differences in serum lipid metabolites in 61 dogs fed an animal-based (MEAT) or plant-based (PLANT) diet for 3 months.

**Metabolites (**μ**M)**		**Time (week)**	**MEAT (n30)**		**PLANT (n31)**		**P_Diet_**	**P_Time_**	**P_DietxTime_**
			**MSC**	**SEM**	**MSC**	**SEM**			
Phosphatidyl-cholines	PC aa C32:2	0	3.98	0.09	3.78^A^	0.10	<0.001	>0.1	<0.001
		12	3.90^c^		2.99^Bd^				
	PC aa C36:0	0	18.62	0.47	17.90^A^	0.62	0.021	>0.1	<0.001
		12	17.99^c^		20.97^Bd^				
	PC aa C38:0	0	5.20^A^	0.20	5.21^A^	0.27	0.002	0.013	<0.001
		12	4.86^Bc^		6.25^Bd^				
	PC aa C40:1	0	0.77	0.02	0.80^A^	0.03	0.008	>0.1	<0.001
		12	0.75^c^		0.92^Bd^				
	PC aa C40:2	0	1.23	0.03	1.23^A^	0.04	0.026	>0.1	0.002
		12	1.21^c^		1.42^Bd^				
	PC aa C40:6	0	89.02^A^	3.82	82.37^A^	5.12	0.014	0.040	0.001
		12	81.97^Bc^		97.45^Bd^				
	PC ae C36:0	0	3.35	0.11	3.30^A^	0.12	0.029	>0.1	<0.001
		12	3.21^c^		2.73^Bd^				
	PC ae C40:6	0	10.23^A^	0.33	10.55^A^	0.47	0.003	0.010	0.001
		12	9.72^Bc^		12.10^Bd^				
	LPC a C16:0	0	69.19	1.57	69.91^A^	1.90	<0.001	>0.1	<0.001
		12	66.97^c^		51.62^Bd^				
	LPC a C17:0	0	1.79	0.05	1.79^A^	0.06	0.035	0.100	<0.001
		12	1.71^c^		1.95^Bd^				
	LPC a C18:0	0	54.79	1.40	56.14^A^	1.55	>0.1	0.002	>0.1
		12	53.55		51.14^B^				
	LPC a C18:1	0	22.82	0.60	23.72^A^	1.14	<0.001	>0.1	<0.001
		12	21.87^c^		34.44^Bd^				
	LPC a C18:2	0	26.28	1.01	28.78^A^	1.01	>0.1	>0.1	0.009
		12	25.68		24.76^B^				
	LPC a C20:4	0	12.50	0.38	12.65^A^	0.38	0.082	>0.1	0.001
		12	12.25		11.01^B^				
	LPC a C24:0	0	0.17	0.01	0.19^A^	0.01	<0.001	>0.1	<0.001
		12	0.17^c^		0.25^Bd^				
Sphingomyelins	SM C16:0	0	176.56	5.18	172.48^A^	4.47	<0.001	>0.1	<0.001
		12	169.89^c^		135.35^Bd^				
	SM C16:1	0	15.74	0.49	15.04^A^	0.43	0.004	0.100	<0.001
		12	15.07^c^		12.30^Bd^				
	SM C18:0	0	40.54	1.16	36.86^A^	1.19	<0.001	>0.1	<0.001
		12	39.16^c^		27.25^Bd^				
	SM C18:1	0	15.53	0.44	14.39^A^	0.46	<0.001	>0.1	<0.001
		12	15.05^c^		11.36^Bd^				
	SM C20:2	0	0.69	0.02	0.69^A^	0.02	0.001	>0.1	<0.001
		12	0.68^c^		0.53^Bd^				
	SMOH C14:1	0	7.62^A^	0.26	7.40^A^	0.26	>0.1	0.012	>0.1
		12	7.11^B^		6.82^B^				
	SMOH C16:1	0	6.66^A^	0.21	5.941^A^	0.19	0.056	0.031	>0.1
		12	6.24^B^		5.29^B^				
	SMOH C22:1	0	20.66	0.58	19.76^A^	0.56	0.016	0.083	0.002
		12	19.77c		16.60^Bd^				
	SMOH C22:2	0	13.88	0.37	13.31^A^	0.40	0.002	0.100	<0.001
		12	13.33^c^		10.75^Bd^				
	SMOH C24:1	0	3.15	0.08	2.98^A^	0.08	0.004	0.100	0.001
		12	3.03^c^		2.51^Bd^				
Carnitines	C0	0	38.12	1.39	36.74^A^	1.28	0.014	>0.1	<0.01
		12	37.33^c^		43.73^Bd^				
	C2	0	3.35	0.17	2.89^A^	0.14	>0.1	>0.1	0.023
		12	3.26		3.44^B^				
	C3	0	0.24	0.0.1	0.2^A^	0.01	>0.1	>0.1	0.013
		12	0.23		0.24 ^B^				
	C4	0	0.13	0.004	0.12^A^	0.003	>0.1	>0.1	0.020
		12	0.13		0.13^B^				
	C5	0	0.17	0.01	0.15^A^	0.01	>0.1	0.001	>0.1
		12	0.18		0.18^B^				
	C14:1	0	0.07	0.004	0.07^A^	0.006	<0.001	>0.1	<0.001
		12	0.06^c^		0.10^Bd^				
	C16	0	0.13	0.004	0.12^A^	0.004	>0.1	0.002	>0.1
		12	0.13		0.10^B^				
	C16:1	0	0.05	0.001	0.05	0.001	>0.1	>0.1	<0.001
		12	0.05		0.05				
	C16:2 (OH)	0	0.020	0.0004	0.019^A^	0.0005	>0.1	0.006	>0.1
		12	0.020		0.022^B^				

MSC, mean serum concentration; PC, Phosphatidylcholine; LPC, lysophosphatidylcholine; SM, sphingomyelin; C0, carnitine; C2, acetylcarnitine; C3, propionylcarnitine; C4, butyrylcarnitine; C5, valerylcarnitine; C14:1, tetradecenoyl carnitine; C16, hexadecanoylcarnitine; C16:1, dexadecenoylcarnitine; C16:2-OH, hydroxyhexadecadienylcarnitine.

Rows or columns with no superscript indicates no significant differences for the measured parameter.

Down a column, different upper-case superscript letters (A, B) denote significant differences (*P* < 0.05) following a Tukey's *post hoc* analysis within groups (MEAT or PLANT) over time (week 0 to week 12).

Across a row, different lower-case letters (c, d) denote significant differences (*P* < 0.05) following a Tukey's *post hoc* analysis between groups (MEAT or PLANT) at one time point (week 0 or week 12).

### TMIC one-carbon and folate metabolism pathway

**Figure 8:**
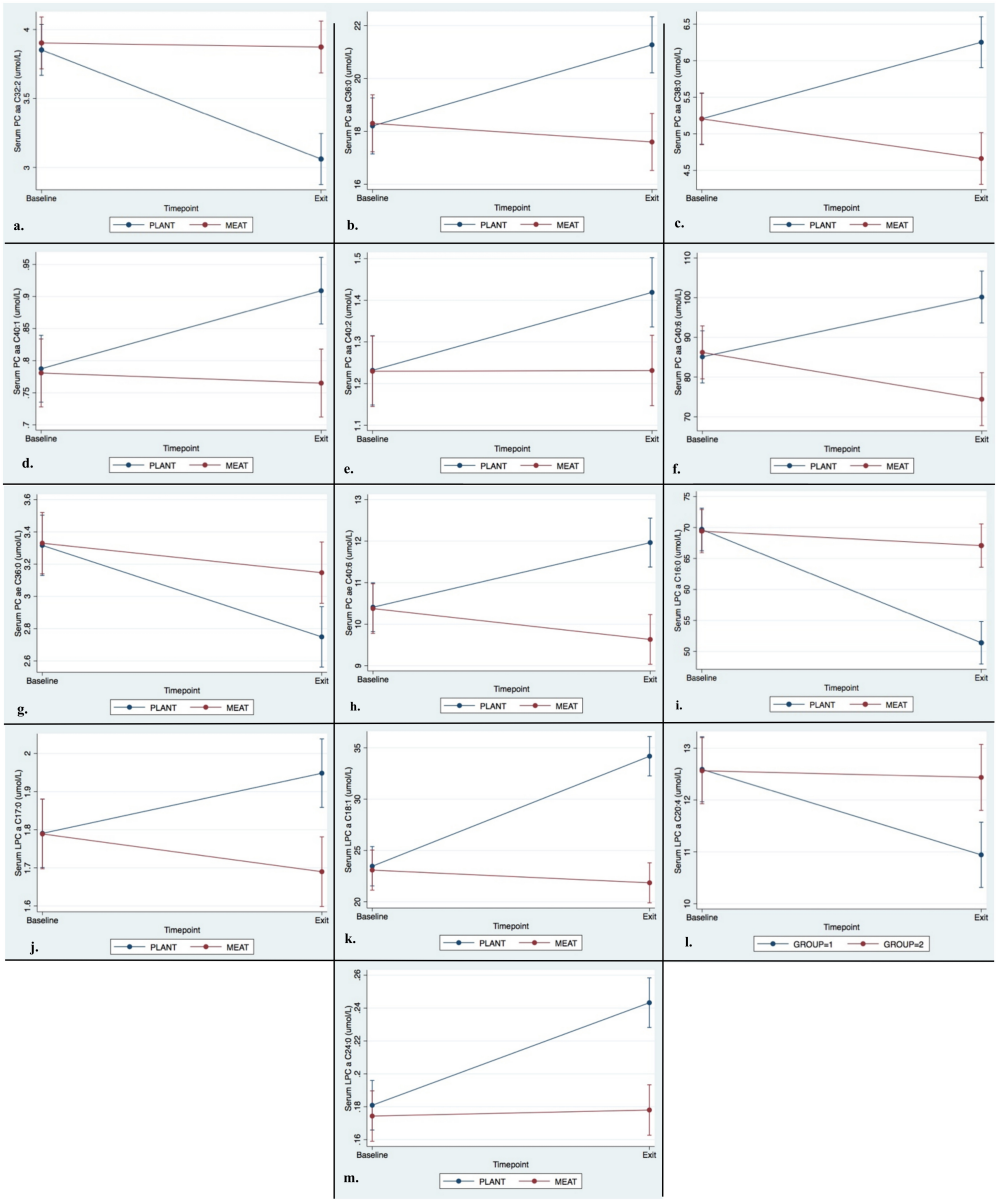
Phosphatidylcholines **(a)** PC aa C32:3, **(b)** PC aa C36:0, **(c)** PC aa C38:0, **(d)** PC aa C40:1, **(e)** PC aa C40:2, **(f)** PC aa C40:6, **(g)** PC ae C36:0, **(h)** PC ae C40:6, **(i)** LPC a C16:0, **(j)** LPC a C17:0, **(k)** LPC a C18:1, **(l)** LPC a C20:4, **(m)** LPC a C24:0 with significant differences between diet groups at the exit timepoint detected by repeated measures mixed modeling in 61 dogs fed an animal-based (MEAT) or plant-based Q23 (PLANT) diet for 3 months.

A separate metabolomic analysis, LC-MRM/MS, was performed to measure 31 one-carbon and folate metabolism pathway metabolites (TMIC One-carbon and Folate Metabolism Pathway). Three (thiamine, pyridoxamine, and hypotaurine) were below the detection level and excluded, leaving a total of 28 folate pathway and 1C metabolites available for statistical evaluation. Based on univariate mixed modeling, eight analytes (5-MTF, betaine, cobalamin, cysteine, cystine, dcSAM, homocysteine, and MMA) were determined to be affected (Table 4, Supplementary Table 7), with three (dcSAM, betaine, and MMA) significantly different between diet groups at the exit timepoint (Figure 8). Betaine and MMA were higher in PLANT, while dcSAM was lower.

**Table 4:** Significant differences in folate and one-carbon metabolites in 61 dogs fed an animal-based (MEAT) or plant-based (PLANT) diet for 3 months.

**Metabolites (μM)**	**Time (week)**	**MEAT (n30)**		**PLANT (n31)**		**P_Diet_**	**P_Time_**	**P_DietxTime_**
		**MSC**	**SEM**	**MSC**	**SEM**			
5-MTHF	0	0.012	0.0004	0.011^A^	0.0006	0.083	0.006	>0.1
	12	0.012		0.009^B^				
Betaine	0	92.86^A^	3.57	95.11	3.15	>0.1	>0.1	<0.001
	12	95.37^B^		96.24				
Cysteine	0	2.65^A^	0.39	3.46^A^	0.45	>0.1	0.015	>0.1
	12	2.93^B^		3.33^B^				
Cystine	0	27.74^A^	0.98	23.36^A^	0.78	>0.1	0.001	>0.1
	12	26.66^B^		24.86^B^				
Cobalamin	0	1.9e-5^A^	1.6e-6	1.8e-5	1.6e-6	>0.1	<0.001	>0.1
	12	2.0e-5^B^		1.8e-5				
dcSAM	0	0.026	0.001	0.027^A^	0.001	0.024	0.100	<0.001
	12	0.027^c^		0.025^Bd^				
Homocysteine	0	9.19^A^	0.19	9.19^A^	0.20	>0.1	<0.001	>0.1
	12	9.05^B^		9.09^B^				
Methylmalonic acid	0	0.49	0.018	0.47^A^	0.025	0.065	>0.1	<0.001
	12	0.48^c^		0.59^Bd^				

MSC, mean serum concentration; 5-MTHF, 5-methyltetrahydrofolate; dcSAM, S-adenosyl-methioninamine; MMA, methylmalonic acid.

Rows or columns with no superscript indicates no significant differences for the measured parameter.

Down a column, different upper-case superscript letters (A, B) denote significant differences (*P* < 0.05) following a Tukey's *post hoc* analysis within groups (MEAT or PLANT) over time (week 0 to week 12).

Across a row, different lower-case letters (c, d) denote significant differences (*P* < 0.05) following a Tukey's *post hoc* analysis between groups (MEAT or PLANT) at one time point (week 0 or week 12).

### Fatty acid analysis

**Figure 9:**
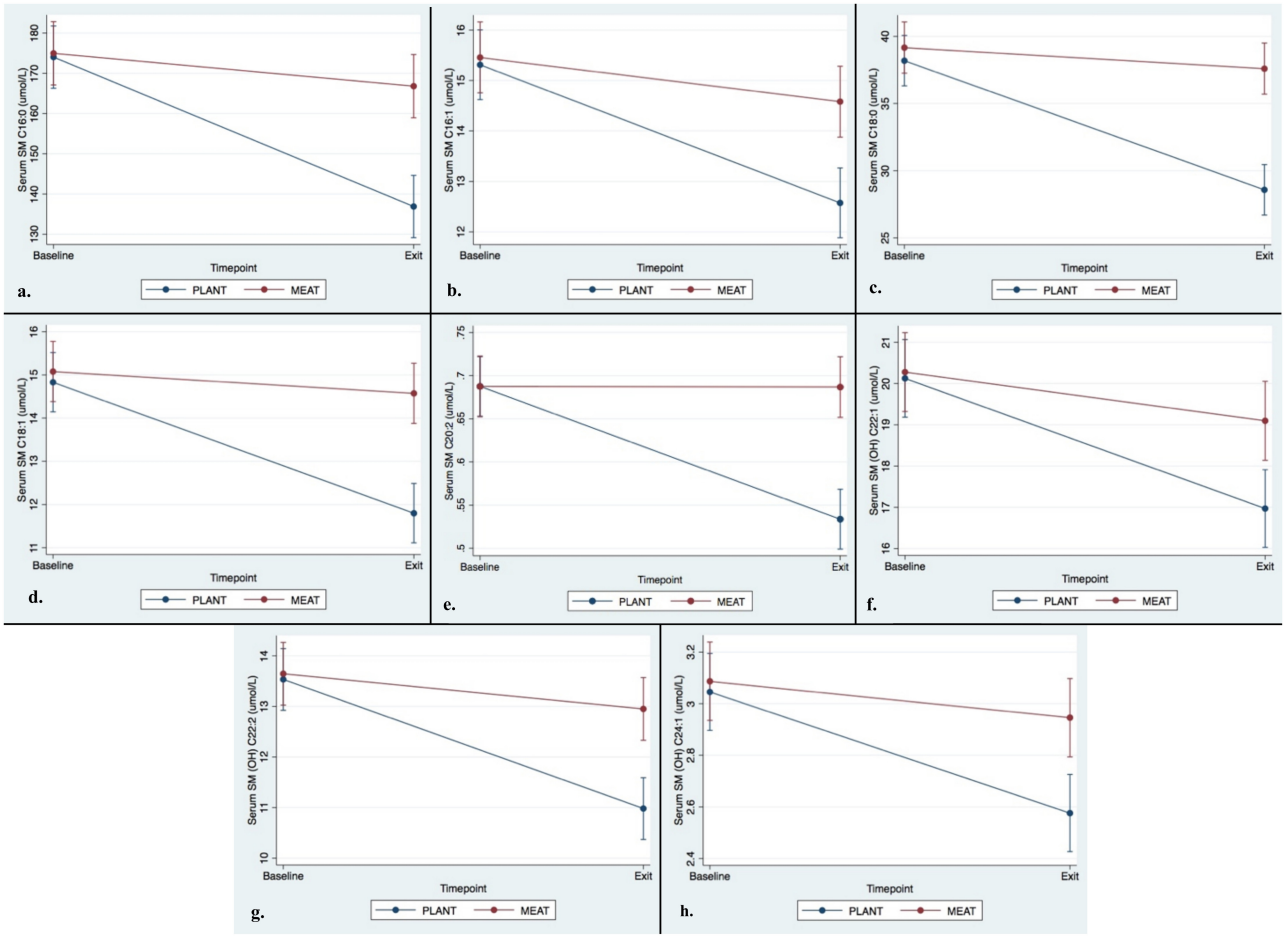
Sphingomyelins **(a)** SM C16:0, **(b)** SM C16:1, **(c)** SM C18:0, **(d)** SM C18:1, **(e)** SM C20:2, **(f)** SM(OH) C22:1, **(g)** SM(OH) C22:2, **(h)** SM(OH) C24:1 with significant differences between diet groups at the exit timepoint detected by repeated measures mixed modeling in 61 dogs fed an animal-based (MEAT) or plant-based (PLANT) diet for 3 months.

**Figure 10:**
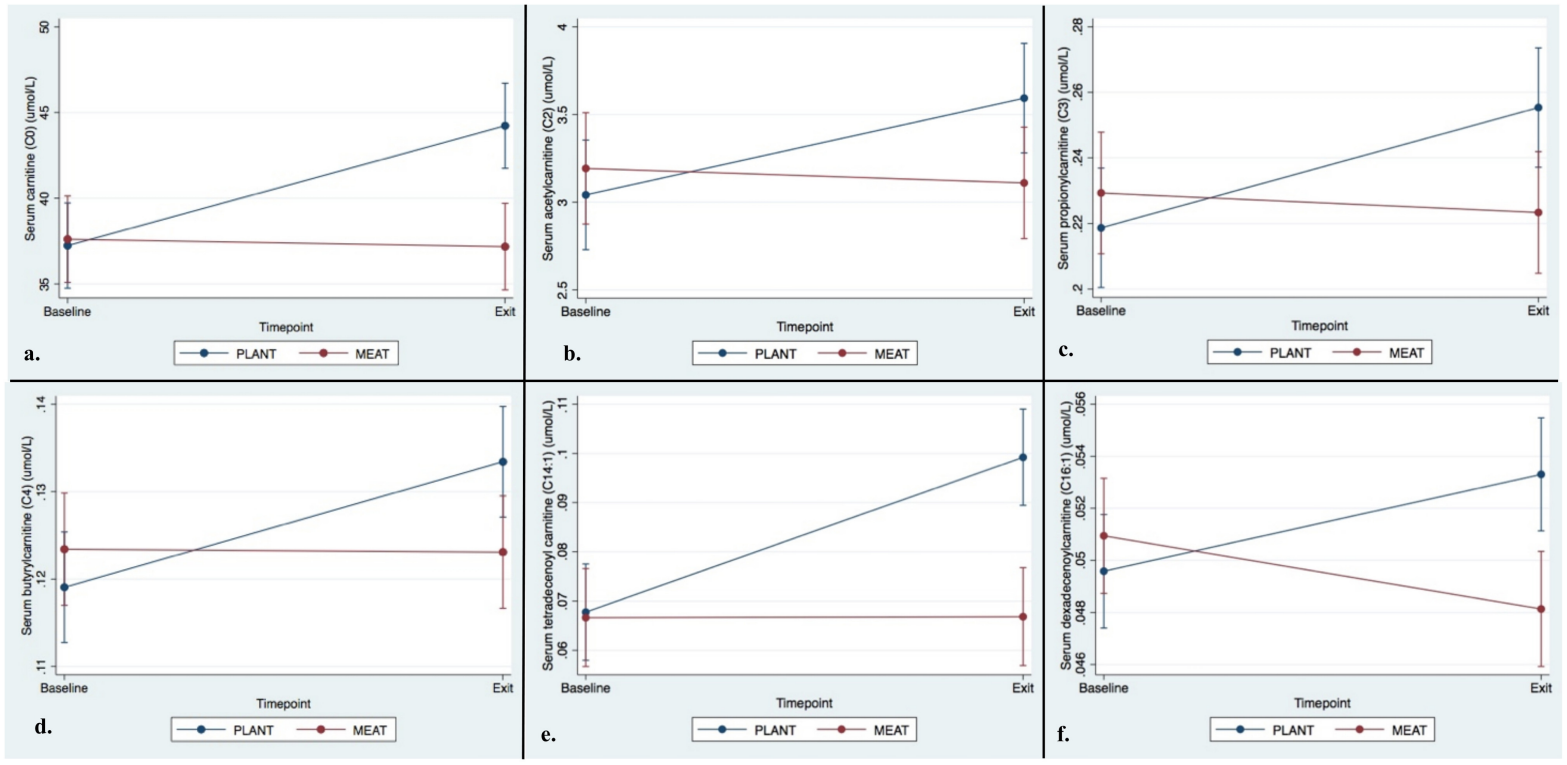
Carnitine **(a)** and acylcarnitines **(b)** C2, **(c)** C3, **(d)** C4, **(e)** C14:1, **(f)** C16:1 with significant differences between diet groups at the exit timepoint detected by repeated measures mixed modeling in 61 dogs fed an animal-based (MEAT) or plant-based (PLANT) diet for 3 months.

**Figure 11:**
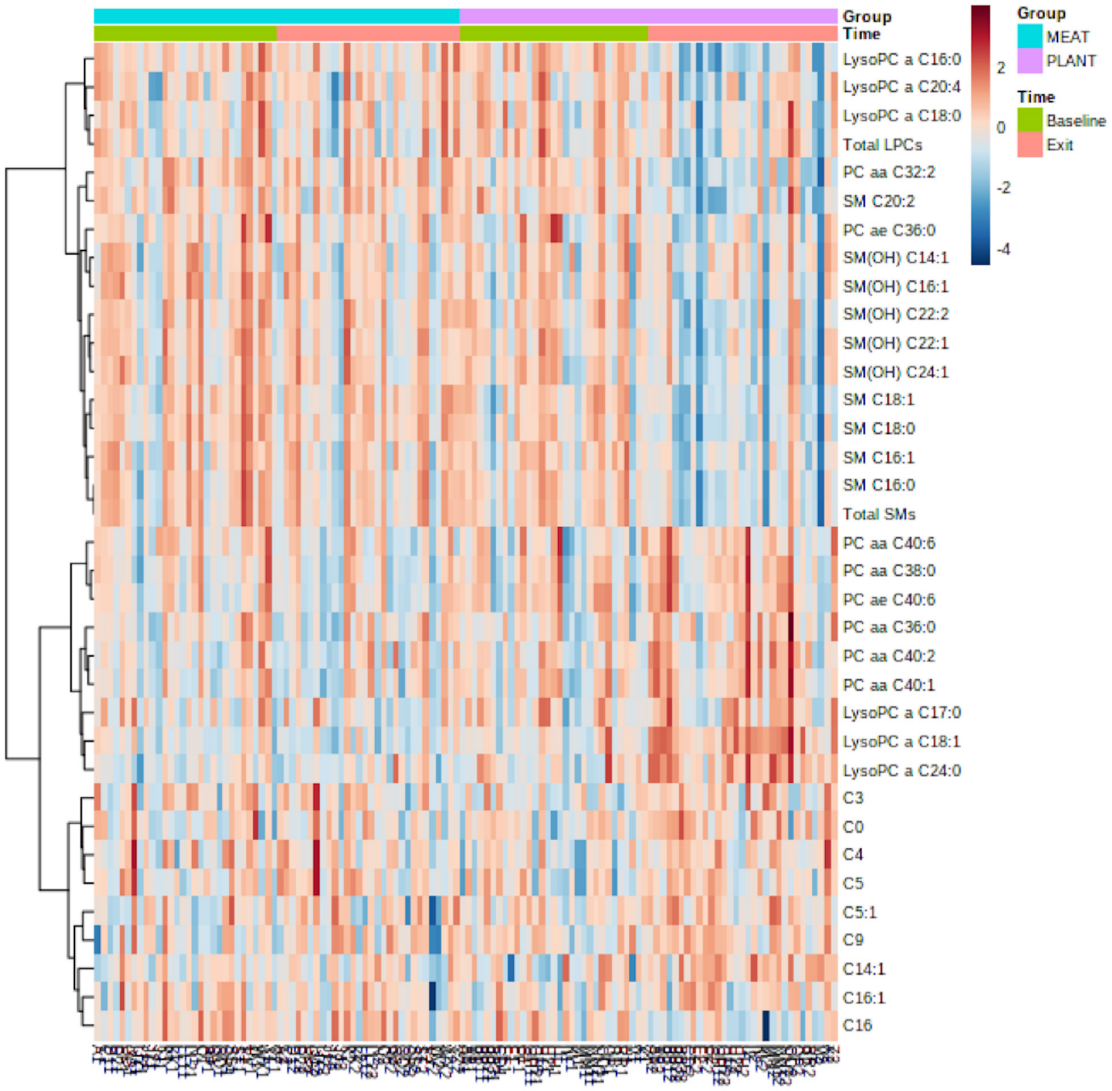
Two-way heatmap of significant lipid metabolites in 61 dogs fed an animal-based (MEAT) or plant-based (PLANT) diet for 3 months, based on repeated measures mixed modeling.

A total of 67 FA were measured by gas chromatography, with 6 below the detection limit, leaving 61 FA for analysis. On univariate repeated measures mixed modeling, eight saturated FA (14:0, 16:0, 18:0, 21:0, 22:0, 23:0, 24:0, and total saturated FA), eight monounsaturated (MU) FA (16:1c9, 18:1c9, 18:1c11, 20:1c11, 18:1t6-8, 18:1t12, 18:1t13&14, 18:1t16, and 24:1n9), six polyunsaturated (PU) FA (18:2n6, 18:3n6, 20:3n6, 20:4n6, 22:4n6, and 22:6n3), the ratio of omega (ω) 6:3 FA, and total FA were found to be affected (Table 5, Supplementary Table 8). Fatty acids significantly different between diet groups at the exit time point are depicted in Figures 9, 10. Among the saturated FA, 14:0, 16:0, 18:0, and 23:0 were lower in PLANT, 22:0 and 24:0 were higher. Monounsaturated trans 18:1t13&14 and 18:1t16 were lower in PLANT, 18:1t6-8 was higher, while MUFA 16:1c9 and 18:1c11 were lower in PLANT, and 18:1c9 and 20:1c11 were higher. The ω-9 MUFA 24:1n9 and all of the differing ω-6 PUFA were lower in PLANT (18:2n6, 18:3n6, 20:3n6, 20:4n6, 22:4n6), while the ω-3 PUFA 22:6n3 was higher. All fatty acids affected by time, diet, or the interaction of time and diet, as identified on mixed modeling, were included for further analyses using the MetaboAnalyst^b^ platform. A heatmap was generated to visualize differences between the diet groups and timepoints (Figure 11).

**Table 5:** Significant differences in serum fatty acids in 61 dogs fed an animal-based (MEAT) or plant-based (PLANT) diet for 3 months.

**Fatty acids (mg/ml)**	**Time (week)**	**MEAT (n30)**		**PLANT (n31)**		**P_Diet_**	**P_Time_**	**P_DietxTime_**
		**MSC**	**SEM**	**MSC**	**SEM**			
**Saturated**								
14:0	0	10.46	0.34	10.30^A^	0.42	0.032	>0.1	<0.001
	12	10.24^c^		9.01^Bd^				
16:0	0	700.65	15.93	667.79^A^	20.59	<0.001	>0.1	<0.001
	12	720.72^c^		463.53^Bd^				
18:0	0	816.16	20.79	780.56^A^	20.71	<0.001	>0.1	<0.001
	12	836.67^c^		666.74^Bd^				
21:0	0	3.70^Ac^	0.21	4.02^Ad^	0.26	0.005	0.001	>0.1
	12	4.14^Bc^		5.41^Bd^				
22:0	0	12.91	0.60	13.96^A^	0.69	<0.001	>0.1	<0.001
	12	13.78^c^		20.02^Bd^				
23:0	0	9.12	0.37	9.21^A^	0.30	0.003	>0.1	<0.001
	12	9.34		7.37^Bd^				
24:0	0	12.77	0.50	14.30^A^	0.49	<0.001	>0.1	0.001
	12	13.58^c^		16.95^Bd^				
Total	0	1600.97	37.02	1534.40^A^	40.40	0.001	>0.1	<0.001
	12	1643.79^c^		1226.74^Bd^				
**Monounsaturated** ***cis***								
16:1c9	0	49.46^c^	1.73	43.60^Ad^	1.97	0.047	0.096	<0.001
	12	52.65^c^		25.80^Bd^				
18:1c9	0	559.33	14.11	520.43^A^	29.75	<0.001	>0.1	<0.001
	12	564.29^c^		816.53^Bd^				
18:1c11	0	126.02	3.35	117.13^A^	3.83	<0.001	0.100	<0.001
	12	128.57c		95.49^Bd^				
20:1c11	0	9.18	0.38	8.81^A^	0.74	<0.001	>0.1	<0.001
	12	9.43^c^		15.12^Bd^				
24:1n9	0	33.08	1.12	32.66^A^	1.10	<0.001	>0.1	<0.001
	12	32.97^c^		24.52^Bd^				
**Monounsaturated** ***trans***								
18:1t6-8	0	3.20	0.15	2.86^A^	0.23	<0.001	>0.1	<0.001
	12	2.85^c^		4.68^Bd^				
18:1t12	0	1.87	0.10	1.85^A^	0.08	0.035	0.010	0.05
	12	1.98^c^		1.52^Bd^				
18:1t13&14	0	5.80	0.46	5.41^A^	0.29	<0.001	>0.1	<0.001
	12	6.84^c^		4.10^Bd^				
18:1t16	0	3.19	0.14	2.90^A^	0.14	0.005	>0.1	0.001
	12	3.18^c^		2.36^Bd^				
**Polyunsaturated**								
18:2ω6	0	1324.37	37.30	1288.85^A^	40.57	<0.001	>0.1	<0.001
	12	1363.73^c^		1020.47^Bd^				
18:3ω6	0	15.26	0.72	17.22^A^	0.70	<0.001	>0.1	0.001
	12	16.14^c^		12.45^Bd^				
20:3ω6	0	46.71^c^	1.86	40.11^Ad^	1.42	0.021	>0.1	<0.001
	12	46.71^c^		34.19^Bd^				
20:4ω6	0	881.21^c^	27.18	853.23^A^	26.45	<0.001	0.031	<0.001
	12	918.95^c^		647.34^Bd^				
22:4ω6	0	42.96^Ac^	3.74	42.45^A^	3.18	<0.001	<0.001	>0,1
	12	54.94^B^		28.88^Bd^				
22:5ω6	0	11.71	0.46	10.37^A^	0.46	>0.1	0.001	>0.1
	12	11.88		13.11^B^				
22:6ω3	0	65.54	3.16	64.94^A^	3.92	0.013	>0.1	0.010
	12	59.50^c^		74.62^Bd^				
ω6:ω3	0	12.03	0.34	12.12^A^	0.40	<0.001	0.002	<0.001
	12	13.27^c^		9.06^Bd^				
Total	0	5015.01	121.37	4824.03^A^	128.05	0.001	0.081	<0.001
	12	5161.82^c^		4282.03^Bd^				

MSC, mean serum concentration; ω, omega.

Rows or columns with no superscript indicate no significant differences for the measured parameter.

Down a column, different upper-case superscript letters (A, B) denote significant differences (*P* < 0.05) following a Tukey's *post hoc* analysis within groups (MEAT or PLANT) over time (week 0 to week 12).

Across a row, different lower-case letters (c, d) denote significant differences (*P* < 0.05) following a Tukey's *post hoc* analysis between groups (MEAT or PLANT) at one time point (week 0 or week 12).

## Discussion

Serum metabolomics, comprising protein and amino acid derivatives, carbohydrate and TCA cycle intermediates, folate pathway and 1C donors, and fatty acid and lipid metabolites, were compared between dogs fed extruded PLANT or MEAT diets, using three analytic assays: DI/LC-MS/MS, LC-MRM-MS, and gas chromatography.

Though concerns have been raised regarding protein quantity and quality in plant-based diets for dogs (5, 7, 11), the present study noted fewer alterations to protein and amino acid metabolism than expected, and, with the exception of isoleucine, serum concentrations of all amino acids, derivatives, and protein metabolites were within the ranges reported for apparently healthy adult dogs, where such ranges have been defined (51). There was only a small difference in total protein content between the MEAT and PLANT diets, with MEAT providing slightly more than PLANT (17), and neither dry matter nor crude protein total tract apparent digestibility differed between the two diets (49).

A reduction in serum isoleucine was demonstrated by dogs fed PLANT, despite a higher content of isoleucine in PLANT compared to MEAT, suggesting potential for decreased absorption or bioavailability, or increased intracellular flux, tissue accumulation, and/or metabolism or degradation. Similarly, dietary valine content was higher in PLANT than in MEAT, and serum valine levels also decreased in the PLANT dogs, though they stayed within the reference range. Both isoleucine and valine are branched-chain amino acids (BCAA), and these differences in serum concentrations may suggest a difference in BCAA metabolism in dogs fed PLANT. The main roles of isoleucine and valine in the body are as protein constituents, with documented signs of deficiency being depression in food intake, weight loss, and, for isoleucine only, a negative nitrogen balance, in immature beagles (52–54). In the present study, dogs in both groups maintained food intake, body weight, and body composition throughout the study (17), suggesting that, although PLANT serum BCAA concentrations were lower than the MEAT group, AA provision and metabolism were sufficient to maintain protein metabolism without detectable differences in stored protein catabolism. Supporting this was a concurrent reduction in serum 3-methylhistidine, a metabolite formed from post-translational methylation of histidine in muscle proteins, which is released when muscle protein is catabolized (55, 56). This, along with a lack of increase in beta-hydroxybutyric acid, a degradation metabolite of BCAA, suggests no increase in protein turnover in PLANT-fed dogs.

Creatinine is a protein metabolite produced from creatine and phosphocreatine and excreted with little reabsorption or secretion through the kidneys. Endogenous creatinine synthesis from creatine has been reported to be closely associated with lean body mass, especially skeletal muscle, with little influence of the quantity of total dietary protein intake in healthy dogs (57–59). However, creatinine may also be obtained from a diet containing animal tissues, especially muscle (meat), as protein sources (60). Changes in serum creatinine concentrations may thus relate to differences in dietary creatine or creatinine, changes in muscle mass or protein catabolism, and/or renal clearance (57, 59, 61). In the PLANT group, serum creatinine decreased to become lower than MEAT by the exit timepoint, and fecal creatinine excretion was also lower (41). Serum creatine levels were not different between PLANT and MEAT, though excretion of creatine in the feces was higher in the PLANT group (41). As noted, bodyweight, BCS, and body composition were maintained in both groups (17), thus the reduction in serum creatinine was not considered consistent with a reduction in muscle mass. This suggests that either dietary creatinine intake was reduced, protein catabolism was reduced, and/or renal clearance was improved. Though levels of creatinine were not measured in the diet, it is likely that intake was lower in the dogs fed PLANT as opposed to MEAT, due to the lack of creatinine in plant materials and abundance of creatine and creatinine in heat-processed animal tissues (60). Ingestion of cooked meat, but not vegetarian meals, increases serum creatinine levels postprandially in humans for at least 3–4 h (62, 63). In this study, all dogs were fasted prior to blood collection at each timepoint, negating post-prandial effects. Pre-prandial serum creatine and plasma creatinine levels do not appear to be affected by dietary creatine intake (64, 65). This could suggest that the decreased serum creatinine concentration in dogs fed PLANT may thus be attributable to reduced protein catabolism or increased renal clearance. Reduced protein turnover could be in agreement with the concurrent reduction in 3-methylhistidine, as mentioned. In humans, supplementation with dietary fiber has been demonstrated to reduce blood urea nitrogen and serum creatinine and improve glomerular filtration (66), so these findings may be explained by the higher total dietary fiber intake in the PLANT diet. Further research is thus warranted to investigate protein conservation and renal function in dogs fed a plant-based diet.

Hydroxyproline, a derivative from post-translational modification of proline by prolyl hydroxylase, is the major component of collagen and plays an essential role in the quaternary triple helix structure (67, 68). Given the abundance of hydroxyproline in animal tissues (69), lower serum levels of trans-hydroxyproline in the PLANT group can likely be explained by lower dietary intake, although hydroxyproline concentration in the trial diets was not measured. Hydroxyproline is also related to glycine turnover (69), and it is possible that the PLANT diet may have had lower glycine than the MEAT diet. Lower levels of glycine were also detected in the PLANT group. Potentially, upregulation of the hydroxyproline to glycine pathway may also have contributed to lower serum levels of hydroxyproline. Fecal trans-hydroxyproline was also lower in the PLANT group (41).

Arginine content of the PLANT diet was lower than MEAT, though serum arginine concentrations did not differ between dogs fed PLANT or MEAT. However, dogs fed PLANT had lower serum ornithine and higher acetylornithine, potentially indicating increased acetylation of ornithine for *de novo* synthesis of arginine. Additionally, in humans, serum acetylornithine has been demonstrated to be a marker of vegetable intake (70), which could be the case in dogs as well, considering the higher vegetable content of PLANT than MEAT.

The sulfur amino acids methionine and cysteine have been a recent area of research in canine nutrition, spurred by concern for potential dietary-associated dilated cardiomyopathy secondary to insufficiency of taurine. The expected alterations in sulfur amino acids and their metabolites were not detected, with no differences observed in methionine, cysteine, cystine, homocysteine, or taurine between diet groups. Furthermore, concentrations of serum folate and most of the folate pathway metabolites did not differ between dogs fed the PLANT or MEAT diet, indicating little difference in 1C metabolism between the two groups.

Of the 1C pathway metabolites, only S-adenosylmethioninamine (dcSAM), betaine, and methylmalonic acid differed. dcSAM, the decarboxylated metabolite of S-adenosylmethionine (SAMe), is the precursor for biosynthesis of the polyamines spermine and spermidine, from arginine, via ornithine and putrescine (71, 72). The main roles of polyamines are as intracellular antioxidants and anti-inflammatory agents, though they may also play a role in gene methylation and cell turnover (72). Decreases in dcSAM concentrations have been associated with suppression of aberrant methylation (72). Possibly, dogs fed PLANT had altered polyamine metabolism, resulting in lower decarboxylation of SAMe to dcSAM, though serum SAMe, spermine, and spermidine concentrations did not differ between diet groups. Microbes also possess dcSAM, with spermine and spermidine also associated with microbial degradation of proteins and free amino acids in food items (73). Given the microbial protein degradation that may occur in raw ingredients pre-processing, animal-based protein sources (meats) may contribute to a higher intake of dcSAM (73) as opposed to plant proteins. Endogenous protein fermentation may also have been lower in dogs fed PLANT, as suggested by a reduction in the relative abundance of protein-fermenting Fusobacteria, compared to the dogs fed MEAT (50). However, evidence of altered protein fermentation was not found when fecal metabolites were measured (41).

Betaine, which can be derived from the diet or endogenously synthesized from choline, functions in the 1C-pathway to re-methylate homocysteine to methionine, without utilizing 5-methyl-tetrahydrofolate. Elevated concentrations of betaine have been found in dogs fed methionine-restricted diets (74). As already mentioned, sulfur amino acid metabolism appeared largely undisturbed in the PLANT-fed dogs, with no differences in methionine concentrations detected between groups. Potentially, the elevated betaine could indicate increased recycling of methionine via methylation of homocysteine, although homocysteine concentrations were unaffected, unlike dogs fed methionine-restricted diets, where elevated betaine was found in conjunction with decreased homocysteine (74). Although PLANT contained less methionine than MEAT, dietary provision exceeded minimum recommendations, and thus the shift in metabolism may have reflected the change in dietary provision without a need to compensate for inadequate intake. These findings are in contrast with those reported by Cavanaugh et al. (75), who found a reduction in plasma choline and betaine in dogs fed a plant-based diet, though the plant-based diet contained slightly less choline and markedly less betaine than the comparison meat-based diet. In the present study, betaine concentrations were not measured in the experimental diets, though choline content was slightly higher in the PLANT diet, potentially contributing to higher circulating betaine levels.

Methylmalonic acid (MMA) is a sensitive indicator of cobalamin deficiency, as it accumulates when cobalamin-dependent enzymatic reactions are impaired (76, 77). Mean serum MMA in the PLANT-fed dogs was higher than in the MEAT-fed dogs at the exit timepoint. Yet, the serum MMA concentration in the PLANT group was still within the low end of the reference interval for healthy dogs with normal cobalamin status (78) and serum cobalamin concentrations were also within normal range for healthy dogs and did not differ between diet groups. Increased MMA concentrations have been demonstrated in dogs and cats with normal serum cobalamin concentrations, prompting a hypothesis of cellular cobalamin deficiency and impaired cobalamin-dependent metabolism in the face of normal serum cobalamin status (78, 79). However, the concentrations of MMA reported in dogs with hypocobalaminemia or cellular cobalamin deficiency were ten-fold higher than the MMA reported in the dogs fed PLANT in this trial. Thus, cellular cobalamin deficiency is also unlikely to explain the changes in MMA. The cobalamin content in PLANT was half the concentration of MEAT (17); potentially, despite maintenance of serum cobalamin intake, the increase in serum MMA could be associated with comparatively lower dietary cobalamin intake, despite sufficient provision.

One third of FA and one third of lipid metabolites differed between diet groups at the end of the trial. Many of the serum lipid profile changes could be explained by differences in the fatty acid composition of the diets (Supplementary Table 4), consistent with other studies (38, 39). Dietary provision of shorter-chain SFA (14:0, 16:0, 18:0) was lower in PLANT, while longer SFA (22:0 and 24:0) were higher, corresponding mostly with the changes in the serum profile. Similarly, the serum profiles of saturated fatty acids and cis-MUFA matched the PLANT dietary fatty acid composition. Of note, total serum lipid concentrations were significantly lower in the PLANT group, yet the PLANT diet provided slightly more fat than the MEAT diet. This finding, combined with the lower cholesterol level in PLANT previously documented (17), warrants further investigation into the potential for lipid-lowering effects of plant-based diets in dogs.

Changes in serum ω-6 and ω-3 PUFA concentrations also matched dietary content, with PLANT containing lower concentrations of ω-6 and higher concentrations of ω-3 PUFA, attributable to the typical fatty acid composition of the fat sources in PLANT {sunflower oil [ω-6] (80), flaxseed [ω-3] (81), marine microalgae [ω-3] (82)} compared to MEAT (chicken fat [ω-6]) (83, 84) in this study (31). In dogs, the long-chain ω-3 PUFA, EPA and DHA, have been noted to provide important health benefits, including reduction of serum cholesterol and triglycerides, improved renal perfusion, reduced inflammation associated with osteoarthritis, obesity, cardiovascular, and inflammatory or immunologic skin disorders (85, 86). The FA profile changes may represent a health benefit in this case for the PLANT compared to the MEAT diets used in this study. However, this is not necessarily a benefit due to the plant-based nature of the diet, *per se*, as oils rich in ω-3PUFA, EPA, and DHA were added to PLANT but not MEAT. The salmon meal in MEAT would have provided some ω-3 PUFA, but it is a more concentrated source of protein than fatty acids. Thus, the finding of increased DHA and the reduced ω-6: ω-3 ratio is not a finding translatable to plant-based diets in general, depending on the fatty acid sources used in the diet formulation.

In addition to the serum fatty acid profile, three classes of lipid metabolites were investigated: PC, LPC, SM, SMOH, and acylcarnitines. Phosphatidylcholines are a group of phospholipids composed of a choline “head” and FA “tails,” and are commonly found in biological membranes, blood lipoproteins, and natural surfactants. These phospholipids play roles in endogenous cholesterol and triacylglyceride transport and cellular signaling, growth, and apoptosis (87, 88). The FA composition of PC impacts cell membrane fluidity and may be influenced by nutrition as well as by disease states (88–91). In this study, phosphatidylcholine aa C40:6 (18:1ω-9/22:5ω-6), with unsaturated FA in both positions, was found to be the most abundant. This was followed by the unsaturated/saturated PC aa 38:6 (22:6ω-3/16:0), doubly saturated PC aa C36:0 (14:0/22:0), and saturated/unsaturated PC ae C40:6 (18:0/22:6ω-3). An increase in most of the phosphatidylcholine species found in the PLANT group may be attributable to the slightly higher concentration of choline in the PLANT than MEAT diets (Table 1). Moreover, many of the FA species of PC elevated in the dogs fed PLANT corresponded with higher concentrations of those FA in PLANT as compared to MEAT, apart from the short-chain saturated FA. In humans, PC concentrations of 16:0, 18:1, 20:5ω-3, 22:5ω-3, and 22:6ω-3 FA were lower in vegans, with higher proportions 18:2ω-6, 20:2ω-6, 20:4ω-6 and 22:4ω-6, and a lipid metabolite profile suggested to be protective against angina pectoris, hyperlipidemia and ischemic heart disease (91). These findings were not replicated in the present study, as the PLANT dogs only had lower PC 16:1n-7, but had higher quantities of PCs with saturated FA 16:0, 18:0, 20:0, 22:0 and unsaturated FA 18:1ω-9, 20:1ω-9, 22:5ω-6, 22:6ω-3 and 24:1ω-9. Hydrolysis of phosphatidylcholines by lysophosphatidylcholine acyltransferase (LPCAT) enzymes results in the production of LPC, which can, in turn, be re-acylated by LPCAT back into PC (92). These lipid molecules are constantly turning over, with the freeing and recombination of PC FA. In the present study, the most prevalent LPCs were 16:0 and 18:0. Lysophosphatidylcholine 18:2, and 18:1 were also found in high concentrations. Interestingly, changes in LPC concentrations did not always match changes in PC concentrations. Despite higher concentration of PC 16:0 and 20:4, PLANT dogs had lower proportions of LPC16:0 and 20:4. This potentially may indicate reduced hydrolysis of these PC in particular. It is possible the increased PC 18:1 and simultaneously increased LPC 18:1 in PLANT dogs represented a greater abundance of 18 carbon MUFA available for PC production, as well as a preference for hydrolysis of PC 18:1 to LPC 18:1. Previously, it has been demonstrated that higher 18:1-CoA concentrations could inhibit the acylation activity of a particular LPCAT enzyme (92), perhaps partially explaining the increase in LPC 18:1 corresponding with the increased PC 18:1.

Sphingomyelins are major components of cell membranes, comprised of a phosphatidylcholine or phosphatidyl ethanolamine linked to a sphingosine ceramide backbone and a FA (32). Sphingomyelin roles in membrane fluidity and cellular signaling appear to be influenced by the level of saturation or desaturation of their FA chains (32, 93). In other species, alterations of SM metabolism have been associated with insulin resistance, obesity, cardiovascular disease, neoplasia, and central nervous system diseases (87). Sphingomyelins with longer saturated FA chains have been associated with reduced risk of heart disease and heart failure in humans in comparison to those with shorter saturated FA chains (94, 95). In the current study, SM 16:0 was the most abundant, followed by SM 18:0, SMOH 22:1, and SM 16:1 and 18:1. The PLANT group had lower concentrations of SMs with short-chain saturated FA C16:0 and 18:0, but also lower concentrations of SMs with unsaturated FA C16:1, 18:1, 20:2, 22:1, and 24:1. This corresponded closely with lower concentrations of 16:0, 18:0, 16:1, and 20:2 FA, though not with the higher 18:1 FA. Few studies have examined SM metabolism in dogs. In one canine study of myxomatous mitral valve disease, lower concentrations of SM 16:0 were associated with slowed progression of disease in dogs fed a cardioprotective diet (32). However, those dogs also had elevated concentrations of SM 20:0, 22:0, and 24:0, which were not measured in the current study. Given the dogs in the present study had no evidence of cardiac disease, the clinical significance of this finding is unknown.

Though carnitine is an amino acid derivative, it has been considered with the lipid metabolites due to its role in fatty acid catabolism. L-carnitine, the mammalian stereoisomer, functions intracellularly to shuttle free FA from the cytosol into the mitochondria for β-oxidation and energy production (96). Endogenous carnitine exists in flux with a pool of various acylcarnitines, the esterified product for fatty acid transportation. Within this pool, carnitine is the predominant metabolite. Carnitine and acylcarnitine homeostasis is carefully balanced by dietary intake, endogenous synthesis, and renal tubular reabsorption (96). Changes in carnitine and acylcarnitine concentrations may thus be a result of dietary changes, mitochondrial function, and renal metabolism. In the PLANT-fed dogs, serum carnitine, individual acylcarnitines, and total carnitine concentrations were higher than the dogs fed MEAT. As plant-based ingredients bring negligible dietary carnitine (96), supplementary carnitine was added to the PLANT diet. It is thus likely that the higher total carnitine concentrations detected in the serum of the PLANT dogs are attributable to the supplemental carnitine added to the diet, though it is unclear if this also explains the differences noted in acylcarnitine proportions. Studies of carnitine metabolism in dogs are scarce, with possible associations between a decreased ratio of short-chain acylcarnitines to free carnitine and chronic enteropathy (97), or between long-chain acylcarnitines and glucose and insulin metabolism (98). In humans, a link between long-chain acylcarnitines and insulin sensitivity has also been demonstrated (99). Given the differences in dietary provision of carnitine in the PLANT and MEAT diets, the physiological significance of these findings is unknown.

Limitations to this study included factors related to the dogs, their environments, and the diets used. As the diet trial was performed using privately owned dogs living in their homes, there was a variety of breeds, ages, sexes, and lifestyles. While this is more representative of pet dogs at large when compared to using colonies of dogs, it does introduce greater variability. With client-owned dogs, there was more flexibility in the timing of procedures, and although each dog was fasted overnight (from 8 p.m.) prior to their appointment, with scheduling private appointments, some dogs were fasted for longer than others, with up to a 4-h difference between individuals. Furthermore, given that the dogs were maintained in their homes, there was limited ability for the research team to monitor and ensure study protocols were adhered to. Diet diaries were kept by the participants, though compliance with diary completion was poor (49). Nevertheless, indicators of adherence to a strictly plant-based diet were consistent among the PLANT group, such as a replacement of serum vitamin D3 markers with D2 (17), giving confidence that the protocol was adhered to adequately. Numerous findings, most notably the alterations in fatty acids and lipid metabolites, were attributed to the difference in dietary nutrient composition. Macronutrient (total protein, fat, and carbohydrates) composition was maintained quite similarly between the diets, with fairly comparable energy density, but the MEAT diet was a commercially available product, while the PLANT diet was formulated to be representative of commercially available products, as opposed to formulating the diets to have the same micronutrient profile. The benefit was to have increased external validity, but the limitation is that alterations in serum metabolomics could be attributable to differences in nutrient quantity, quality, and bioavailability, as opposed to the source ingredients. Although the diets were designed to be isoenergetic and similar in macronutrient profile, the fiber fractions differed markedly due to the difference in ingredients between the diets. Of note, PLANT offered a greater total dietary fiber content with a larger soluble fiber fraction; however, this is characteristic of plant-derived proteins and plant-based diets in general. While these differences in fiber composition had minimal impact on nutrient digestibility (49) and fecal microbiota profiles (50), it is possible that shifts in gut microbial metabolism may have contributed to some of the metabolic alterations seen in the host animals' sera. In particular, serum amino acids, protein metabolites, lipids, and lipid metabolites have, in other species, been demonstrated to be strongly influenced by dietary fiber, particularly soluble fiber (100). Finally, serum metabolomics represent a snapshot in time and do not describe the flux of nutrients. Inferences may be hypothesized, but relationships between nutrients cannot be determined with single timepoint serum analysis.

## Conclusion

Serum metabolomic assessment showed minimal changes in protein and amino acid metabolism in the dogs fed PLANT in comparison to MEAT, and most could be explained by differences in dietary nutrient composition as opposed to ingredient sources. Though in relation to protein and amino acid metabolism, further study of creatinine metabolism, renal function, and oxidative status in dogs fed plant-based diets is warranted. Moreover, the association between eating a plant-based diet and changes in BCAA concentration in dogs warrants further investigation, as there was no determinable explanation for the reduction in serum BCAA observed in this study. Given these findings, this study may present a guide for further research investigating these potential effects of plant-based diets on canine metabolomics, particularly using plant-based and meat-based diets formulated to have more closely comparable micronutrient composition.

## Data Availability

The original contributions presented in the study are publicly available. This data can be found here: Dodd, Sarah A.S.; Adolphe, Jennifer L.; Shoveller, Anna Katherina; Dewey, Cate; Khosa, Deep; Ma, David; Abood, Sarah K.; Verbrugghe, Adronie, 2026, “Data for: Metabolic profiles show few differences in serum amino acid, one-carbon and fatty acid compounds in dogs fed a plantbased (‘vegan’) or meat-based diet”, {https://doi.org/10.5683/SP3/XQHLGB}, Borealis, V1.

## Footnotes

1 Petcurean Go! Solutions Skin + Coat Care Chicken Recipe.

2 MetaboAnalyst 5.0, 2021, http://www.metaboanalyst.ca.

3 Human Metabolome Database, 2021, http://www.hmdb.ca.

4 PubChem, 2021, http://www.pubchem.nlm.nih.gov.
