# Supplementary material for: Metabolic profiles show few differences in serum amino acid, one-carbon, and fatty acid compounds in dogs fed a plant-based (“vegan”) or meat-based diet
Source: Front Vet Sci. 2026 Jun 9;12:1706965. doi: 10.3389/fvets.2025.1706965 (PMC13288764; doi:10.3389/fvets.2025.1706965)
Supplement: Supplementary file 1 [file Table_1.DOCX]

**Supplementary Data**

**Supplementary Table 1**

Metabolites identified and quantified in canine sera samples by DI-LC/MS/MS TMIC PRIME assay (TMIC01KF):

| Creatine | Methionine-sulfoxide | Trimethylamine N-oxide | LysoPC a C17:0 | PC aa C38:6 | C9 |
| --- | --- | --- | --- | --- | --- |
| Glycine | Arginine | Methylhistidine | LysoPC a C18:2 | PC aa C38:0 | C7DC |
| Alanine | Acetyl-ornithine | Homocysteine | LysoPC a C18:1 | PC ae C40:6 | C10:2 |
| Serine | Citrulline | Lactic acid | LysoPC a C18:0 | SM(OH) C24:1 | C10:1 |
| Histamine | Serotonin | beta-Hydroxybutyric acid | LysoPC a C20:4 | PC aa C40:6 | C10 |
| Proline | Tyrosine | alpha-Ketoglutaric acid | LysoPC a C20:3 | PC aa C40:2 | C12:1 |
| Valine | DOPA | Citric acid | LysoPC a C24:0 | PC aa C40:1 | C12 |
| Threonine | Asymmetric dimethylarginine | Butyric acid | LysoPC a C26:1 | C0 | C14:2 |
| Phenylethylamine | Total dimethylarginine | Propionic acid | LysoPC a C26:0 | C2 | C14 |
| Taurine | Tryptophan | HPHPA | LysoPC a C28:1 | C3:1 | C12DC |
| Putrescine | Kynurenine | p-hydroxyhippuric acid | LysoPC a C28:0 | C3 | C14:1OH |
| cis-Hydroxyproline | Carnosine | Succinic acid | SM(OH) C14:1 | C4:1 | C14:1OH |
| trans-Hydroxyproline | Nitro-Tyrosine | Fumaric acid | SM C16:1 | C4 | C16:2 |
| Leucine | Ornithine | Pyruvic acid | SM C16:0 | C3OH | C16:1 |
| Isoleucine | Lysine | Isobutyric acid | SM(OH) C16:1 | C5:1 | C16 |
| Asparagine | Spermidine | Hippuric acid | SM C18:1 | C5 | C16:2OH |
| Aspartic acid | Spermine | Methylmalonic acid | PC aa C32:2 | C4OH | C16:1OH |
| Glutamine | Sarcosine | Homovanillic acid | SM C18:0 | C6:1 | C16OH |
| Glutamic acid | Diacetylspermine | Indole acetic acid | SM C20:2 | C6 | C18:2 |
| Methionine | Tyramine | Uric acid | PC ae C36:0 | C5OH | C18:1 |
| Dopamine | Creatine | Glucose | PC aa C36:6 | CG:1DC | C18 |
| Histidine | Phosphocreatine | LysoPC a C14:0 | PC aa C36:0 | C4DC | C18:1OH |
| alpha-Aminoadipic acid | Betaine | LysoPC a C16:1 | SM(OH) C22:2 | C8 |  |
| Phenylalanine | Choline | LysoPC a C16:0 | SM(OH) C22:1 | C5MDC |  |

**Supplementary Table 2**

One-carbon metabolites identified and quantified in canine sera samples by LC-MRM/MS:

| Folic acid | Glutamic acid | Betaine | Homocystine |
| --- | --- | --- | --- |
| 5-MTHF | Glutamine | Choline | Thiamin |
| DHF | Glycine | Cystathionine | Riboflavin |
| THF | Homoserine | Cysteine | Nicotinamide |
| dcSAM | Methionine | Cystine | Pyridoxamine |
| SAH | Serine | GSH | Pyridoxine |
| SAM | Taurine | GSSG | Hypotaurine |
| Vitamin B12 | Acetylcholine | Homocysteine |  |

**Supplementary Table 3**

Free fatty acids identified and quantified in canine sera samples by GC:

| 12:00 | 18:00 | 18:1c13 | 18:2c9c15 | 18:2c/c CLA2 | 20:4n6 |
| --- | --- | --- | --- | --- | --- |
| 12:1c11 | 18:1t4 | 18:1c14 | 20:00 | 18:2tt CLA | 22:2n6 |
| 14:00 | 18:1t5 | 18:2tt | 18:3n6 | 18 4n3 | 24:00 |
| 14:01 | 18:1t6-8 | 18:2t9t12 | 20:1c5 | 21:00 | 20:5n3 |
| 15:00 | 18:1t9 | 18:2c9t13 | 20:1c8 | 20:2n6 | 24:1n9 |
| 15:1c10 | 18:1t10 | 18:2ct | 20:1c11 | 22:00 | 22:3n3 |
| 16:00 | 18:1t11 | 18:2c9t12 | 18:3n3 | 20:3n9 | 22:4n6 |
| 16:1t9 | 18:1t13&14 | 18:2t9c12 | 18:2c9t11 | 20:3n6 | 22:5n6 |
| 16:1c9 | 18:1c9 | 19:1d10 | 18:2c11t13 CLA | 22:1n9 | 22:5n3 |
| 17:00 | 18:1c11 | 18:2n6 | 18:2t10c12 CLA | 20:3n3 | 22:6n3 |
| 17:1c10 | 18:1c12 | 18:2c9c14 | 18:2c/c CLA1 | 23:00 |  |

**Supplementary Table 4**

Fatty acid composition of the commercial animal-based (MEAT) and experimental plant-based (PLANT) diets fed to client-owned dogs in the diet trial

| Fatty acids | | MEAT | PLANT |
| --- | --- | --- | --- |
|  |  | % as fed | |
| Saturated | Total | 3.61 | 1.57 |
| Monounsaturated *cis* | Total | 5.60 | 10.10 |
| Monounsaturated *trans* | Total | 0.09 | 0.08 |
| Polyunsaturated | Total | 3.31 | 2.46 |
| Omega-3 | 18:3ω3 | 0.08 | 0.16 |
|  | 22:6ω3 | 0.01 | 0.01 |
|  | 20:5ω3 | 0.00 | 0.00 |
| Omega-6 | 18:2ω6 | 0.76 | 0.63 |
|  | 20:3ω6 | 0.01 | 0.00 |
|  | 20:4ω6 | 0.02 | 0.00 |

Supplementary Table 5: Significant results of univariate repeated measures mixed model of time and diet for serum amino acids and protein metabolites in 61 dogs fed an animal-based (MEAT) or plant-based (PLANT) diet for 3 months.

| Interaction | Amino acid metabolite | Diet | Contrast (μM) | 95% CI | P-value |
| --- | --- | --- | --- | --- | --- |
| Time (referent = baseline timepoint) | | | | | |
|  | Arginine | PLANT | -7.94 | -14.983- -0.901 | 0.011 |
|  | Glutamine | PLANT | 54.16 | 8.786-99.537 | 0.019 |
|  | Methionine | PLANT | -4.32 | -7.582- -1.063 | 0.005 |
|  | Proline | PLANT | -11.67 | -20.887- -2.461 | 0.008 |
|  | Cysteine | PLANT | 1.25 | 0.037-2.455 | 0.015 |
|  | Cystine | PLANT | -3.62 | -5.891- -1.358 | 0.003 |
|  | Cystine | MEAT | -4.16 | -6.461- -1.854 | 0.001 |
|  | Homocysteine | PLANT | 0.04 | 0.017-0.064 | 0.001 |
| Diet*Time (referent = PLANT at exit timepoint) | | | | | |
|  | Ornithine | MEAT | 3.67 | 1.814-5.532 | <0.001 |
|  | Glycine | MEAT | 47.90 | 30.314-65.489 | <0.001 |
|  | Isoleucine | MEAT | 6.02 | 3.026-9.024 | <0.001 |
|  | Valine | MEAT | 22.31 | 12.511-32.781 | <0.001 |
|  | Acetylornithine | MEAT | -66.06 | -75.374- -56.753 | <0.001 |
|  | Methylhistidine | MEAT | 14.61 | 10.404-18.824 | <0.001 |
|  | Trans-hydroxyproline | MEAT | 19.85 | 14.155-25.549 | <0.001 |
|  | Serotonin | MEAT | 0.60 | 0.328-0.875 | <0.001 |
|  | Creatinine | MEAT | 17.81 | 11.939-23.683 | <0.001 |
|  | Alpha-aminoadipic acid | MEAT | -0.29 | -0.423- -0.147 | <0.001 |
|  | dcSAM | MEAT | 0.007 | 0.0031-0.0112 | <0.001 |

dcSAM = S-adenosyl-methioninamine

Supplementary Table 6: Significant results of univariate repeated measures mixed model of time and diet for each lipid metabolite in 61 dogs fed an animal-based (MEAT) or plant-based (PLANT) diet for 3 months.

| Interaction | Lipid metabolite | Diet | Contrast (uM) | 95% CI | P |
| --- | --- | --- | --- | --- | --- |
| Time (referent = baseline timepoint) | | | | | |
|  | Phosphatidylcholines |  |  |  |  |
|  | PC aa C38:0 | PLANT | 1.05 | 0.555-1.539 | <0.001 |
|  | PC aa C38:0 | MEAT | -0.54 | -1.043- -0.043 | 0.015 |
|  | PC aa C40:6 | PLANT | 15.08 | 5.822-24.342 | <0.001 |
|  | PC aa C40:6 | MEAT | -11.78 | -21.190- -2.364 | 0.008 |
|  | LPC a C18:0 | PLANT | -5.00 | -8.573- -1.426 | 0.002 |
|  | LPC a C18:2 | PLANT | -4.02 | -7.061- -0.980 | 0.009 |
|  | Sphingomyelins |  |  |  |  |
|  | SMOH C14:1 | MEAT | -0.81 | -1.329- -0.287 | 0.002 |
|  | SMOH C16:1 | PLANT | -0.65 | -1.086- -0.208 | 0.004 |
|  | SMOH C16:1 | MEAT | -0.66 | -1.104- -0.212 | 0.004 |
|  | Carnitines |  |  |  |  |
|  | C5 | PLANT | 0.03 | 0.007-0.051 | 0.001 |
|  | C16 | PLANT | -0.02 | -0.033- -0.009 | 0.001 |
|  | C16:2 (OH) | PLANT | 0.002 | 0.0063-0.0038 | 0.006 |
| Diet*Time (referent = PLANT at exit timepoint) | | | | | |
|  | Phosphatidylcholines |  |  |  |  |
|  | PC aa C32:2 | MEAT | 0.81 | 0.548-1.077 | <0.001 |
|  | PC aa C36:0 | MEAT | -3.67 | -5.188- -2.161 | <0.001 |
|  | PC aa C38:0 | MEAT | -1.59 | -2.086- -1.095 | <0.001 |
|  | PC aa C40:1 | MEAT | -0.14 | -0.218- -0.070 | <0.001 |
|  | PC aa C40:2 | MEAT | -0.19 | -0.306- -0.069 | 0.002 |
|  | PC aa C40:6 | MEAT | -25.74 | -35.098- -16.382 | 0.006 |
|  | PC ae C36:0 | MEAT | 0.40 | 0.131-0.665 | <0.001 |
|  | PC ae C40:6 | MEAT | -2.33 | -43.172- -1.493 | 0.001 |
|  | LPC a C16:0 | MEAT | 15.68 | 10.791-20.575 | <0.001 |
|  | LPC a C17:0 | MEAT | -0.26 | -0.386- -0.130 | <0.001 |
|  | LPC a C18:1 | MEAT | -12.34 | -15.074- -9.600 | <0.001 |
|  | LPC a C20:4 | MEAT | 1.49 | 0.599-2.388 | 0.001 |
|  | LPC a C24:0 | MEAT | -0.07 | -0.087- -0.044 | <0.001 |
|  | Sphingomyelins |  |  |  |  |
|  | SM C16:0 | MEAT | 29.91 | 18.872-40.944 | <0.001 |
|  | SM C16:1 | MEAT | 2.00 | 1.016-2.992 | <0.001 |
|  | SM C18:0 | MEAT | 9.02 | 6.329-11.706 | <0.001 |
|  | SM C18:1 | MEAT | 2.77 | 1.789-3.757 | <0.001 |
|  | SM C20:2 | MEAT | 0.15 | 0.104–0.202 | <0.001 |
|  | SMOH C22:1 | MEAT | 2.13 | 0.782-3.470 | 0.002 |
|  | SMOH C22:2 | MEAT | 1.97 | 1.098-2.840 | <0.001 |
|  | SMOH C24:1 | MEAT | 0.37 | 0.156-0.583 | 0.001 |
|  | Carnitines |  |  |  |  |
|  | C0 | MEAT | -7.05 | -10.593- -3.509 | <0.001 |
|  | C2 | MEAT | -0.48 | -0.931- -0.037 | 0.023 |
|  | C3 | MEAT | -0.03 | -0.058- -0.006 | 0.013 |
|  | C4 | MEAT | -0.01 | -0.019- -0.001 | 0.020 |
|  | C14:1 | MEAT | -0.03 | -0.046- -0.018 | <0.001 |
|  | C16:1 | MEAT | -0.005 | -0.0083- -0.0020 | <0.001 |

MSC = mean serum concentration, PC = Phosphatidylcholine, LPC = lysophosphatidylcholine, SM = sphingomyelin, C0 = carnitine, C2 = acetylcarnitine, C3 = propionylcarnitine, C4 = butyrylcarnitine, C5 = valerylcarnitine, C14:1 = tetradecenoyl carnitine, C16 = hexadecanoylcarnitine, C16:1 = dexadecenoylcarnitine, C16:2-OH = hydroxyhexadecadienylcarnitine

Supplementary Table 7: Significant results of univariate repeated measures mixed model of time and diet for serum folate pathway metabolites in 61 dogs fed an animal-based (MEAT) or plant-based (PLANT) diet for 3 months.

| Interaction | Folate pathway metabolite | Diet | Contrast (uM) | 95% CI | P |
| --- | --- | --- | --- | --- | --- |
| Time (referent = baseline timepoint) | | | | | |
|  | 5-MTHF | PLANT | -0.002 | -0.0031- -0.0002 | 0.006 |
|  | Cobalamin | PLANT | 3.19e-06 | 1.33e-06-5.50e-06 | <0.001 |
|  | Cobalamin | MEAT | 2.87e-06 | 9.75e-07-4.76e-06 | <0.001 |
|  | Cystine | PLANT | -3.62 | -5.891- -1.358 | 0.003 |
|  | Cystine | MEAT | -4.16 | -6.461- -1.854 | 0.001 |
|  | Homocysteine | PLANT | 0.04 | 0.017-0.064 | 0.001 |
| Diet*Time (referent = PLANT at exit timepoint) | | | | | |
|  | dcSAM | MEAT | 0.007 | 0.0031-0.0112 | <0.001 |
|  | Betaine | MEAT | -13.04 | -19.5365- -6.5513 | <0.001 |
|  | MMA | MEAT | -0.12 | -0.180- -0.605 | <0.001 |

5-MTHF = 5-methyltetrahydrofolate, dcSAM = S-adenosyl-methioninamine, MMA = methylmalonic acid

Supplementary Table 8: Significant results of univariate repeated measures mixed model of time and diet for serum fatty acids in 61 dogs fed an animal-based (MEAT) or plant-based (PLANT) diet for 3 months.

| Interaction | Fatty acid | Diet | Contrast (mg/mL) | 95% CI | P |
| --- | --- | --- | --- | --- | --- |
| Time (referent = baseline timepoint) | | | | | |
|  | Saturated | | | | |
|  | 21:0 | PLANT | 1.27 | 0.570-1.964 | 0.001 |
|  | Monounsaturated *trans* | | | | |
|  | 18:1t12 | PLANT | -0.30 | -0.566- -0.034 | 0.010 |
|  | Polyunsaturated |  |  |  |  |
|  | 22:4ω6 | PLANT | -12.55 | -23.350- -1.756 | <0.001 |
|  | 22:4ω6 | MEAT | 14.44 | 3.075-25.814 | <0.001 |
|  | 22:5ω6 | PLANT | 2.34 | 0.970-3.720 | 0.001 |
| Diet*Time (referent = PLANT at exit timepoint) | | | | | |
|  | Total fatty acids | MEAT | 786.73 | 461.463-1112.003 | <0.001 |
|  | Saturated | | | | |
|  | 14:0 | MEAT | 1.62 | 0.599-2.631 | <0.001 |
|  | 16:0 | MEAT | 231.04 | 180.594-281.482 | <0.001 |
|  | 18:0 | MEAT | 138.89 | 86.357-191.425 | <0.001 |
|  | 22:0 | MEAT | -5.44 | -7.347- -3.535 | <0.001 |
|  | 23:0 | MEAT | 2.09 | 1.101-3.072 | <0.001 |
|  | 24:0 | MEAT | -2.63 | -4.192- -1.075 | 0.001 |
|  | Total saturated | MEAT | 360.74 | 257.337-464.141 | <0.001 |
|  | Monounsaturated *trans* | | | | |
|  | 18:1t6-8 | MEAT | -1.61 | -2.241- -0.983 | <0.001 |
|  | 18:1t13&14 | MEAT | 2.56 | 1.179-3.946 | <0.001 |
|  | 18:1t16 | MEAT | 0.60 | 0.139-1.053 | 0.001 |
|  | Monounsaturated *cis* | | | | |
|  | 16:1c9 | MEAT | 23.41 | 17.901-28.924 | <0.001 |
|  | 18:1c9 | MEAT | -229.40 | -293.707- -165.095 | <0.001 |
|  | 18:1c11 | MEAT | 26.88 | 16.783-36.980 | <0.001 |
|  | 20:1c11 | MEAT | -5.12 | -6.866- -3.375 | <0.001 |
|  | 24:1n9 | MEAT | 7.34 | 4.132-10.551 | <0.001 |
|  | Polyunsaturated | | | | |
|  | 18:2ω6 | MEAT | 324.41 | 224.419-424.393 | <0.001 |
|  | 18:3ω6 | MEAT | 4.90 | 2.885-6.911 | 0.001 |
|  | 20:3ω6 | MEAT | 9.15 | 5.040-13.265 | <0.001 |
|  | 20:4ω6 | MEAT | 247.77 | 167.957-327.578 | <0.001 |
|  | 22:4ω6 | MEAT | 20.25 | 8.997-31.498 | <0.001 |
|  | 22:6ω3 | MEAT | -11.63 | -20.249- -3.005 | 0.010 |
|  | ω6:ω3 | MEAT | 3.65 | 2.551-4.747 | <0.001 |

ω = omega

**Supplementary Figure 1**

Bland-Altman plot of differences between canine serum glutamic acid as measured by TMIC PRIME or One-carbon and Folate Metabolism Pathway assays.

**Supplementary Figure 2**

Bland-Altman plot of differences between canine serum glutamine as measured by TMIC PRIME or One-carbon and Folate Metabolism Pathway assays.

**Supplementary Figure 3**

Bland-Altman plot of differences between canine serum glycine as measured by TMIC PRIME or One-carbon and Folate Metabolism Pathway assays.

**Supplementary Figure 4**

Bland-Altman plot of differences between canine serum methionine as measured by TMIC PRIME or One-carbon and Folate Metabolism Pathway assays.

**Supplementary Figure 5**

Bland-Altman plot of differences between canine serum serine as measured by TMIC PRIME or One-carbon and Folate Metabolism Pathway assays.

**Supplementary Figure 6**

Bland-Altman plot of differences between canine serum taurine as measured by TMIC PRIME or One-carbon and Folate Metabolism Pathway assays.

Note: differences in sample preparation methods resulted in numerical differences in measured serum taurine values, though patterns between individuals, group and timepoint were not affected.

**Supplementary Figure 7**

Bland-Altman plot of differences between canine serum betaine as measured by TMIC PRIME or One-carbon and Folate Metabolism Pathway assays.

**Supplementary Figure 8**

Bland-Altman plot of differences between canine serum choline as measured by TMIC PRIME or One-carbon and Folate Metabolism Pathway assays.

**Supplementary Figure 9**

Bland-Altman plot of differences between canine serum homocysteine as measured by TMIC PRIME or One-carbon and Folate Metabolism Pathway assays.

Note: differences in sample preparation methods resulted in numerical differences in measured serum homocysteine values, though patterns between individuals, group and timepoint were not affected.
